# Construct validity of the Assessment of Quality of Life - 6D (AQoL-6D) in community samples

**DOI:** 10.1186/1477-7525-11-61

**Published:** 2013-04-17

**Authors:** Joanne Allen, Kerry J Inder, Terry J Lewin, John R Attia, Brian J Kelly

**Affiliations:** 1 Centre for Translational Neuroscience and Mental Health, University of Newcastle and Hunter New England Health, Newcastle, NSW, Australia; 2 Hunter Medical Research Institute, Newcastle, Australia; 3 Centre for Clinical Epidemiology and Biostatistics, University of Newcastle and Hunter New England Health, Newcastle, NSW, Australia

## Abstract

**Background:**

The Assessment of Quality of Life - 6D scale (AQoL-6D) is a self-report instrument designed to provide a sensitive multidimensional evaluation of health related quality of life. The current paper assesses the construct, concurrent and convergent validity of the AQoL-6D in a combined longitudinal population sample drawn from across urban, regional and remote areas of Australia.

**Methods:**

The AQoL-6D was administered within the Hunter Community Study and the Australian Rural Mental Health Study over time (mean years lag = 3.90, SD = 1.30). Observations with sufficient data were used to confirm the construct validity of the AQoL-6D domains and higher-order structure using confirmatory factor analyses (CFA, N = 7915). The stability of this structure across cohorts and over time was assessed using multi-group CFA. Additionally, the concurrent validity (against the SF-36) and convergent validity of AQoL-6D domains and factors were assessed.

**Results:**

The construct validity of the AQoL-6D domains was considered satisfactory. Two higher-order factors, representing the physical and psychological components of quality of life were identified (CFA model fit: RMSEA = .07, SRMR = .03; TLI = .96, CFI = .98). These factors displayed group and temporal invariance, as well as concurrent and convergent validity against a range of measures. Recommendations for the derivation of summary scores are provided, together with a provisional set of norms.

**Conclusions:**

The AQoL-6D is a useful tool for assessing quality of life impairment in epidemiological cohort studies, both cross-sectionally and over time. It displays appropriate levels of construct, concurrent and convergent validity. Conceptualisation of higher-order factors as representing the physical and psychological aspects of quality of life impairment may increase the sensitivity and appeal of the AQoL-6D, particularly for studies examining predictors of and changes in social and psychological outcomes.

## 

With many countries facing the health care challenges associated with an ageing population, factors associated with quality of life (QoL) and its maintenance are of immediate interest and concern. Quality of life has typically been conceptualised as the perception of physical and psychological wellbeing or functioning, although some formulations have also included environmental and social assets [1]. Self-report instruments measuring QoL have generally acknowledged this multidimensional structure, characterising life quality by means of various domains assessed using single or multiple items^a^; the scope of any particular QoL measure is typically determined by a trade-off between the brevity and sensitivity required.

The Assessment of Quality of Life (AQoL) family of measures share an overarching conceptualisation of QoL in terms of handicap, or the impact of a reported health state on personal functioning and satisfaction within the person’s social context [2]. To date the AQoL family comprises four multidimensional self-report survey instruments, each assessing somewhat differing domains of QoL. While scale construction often involves the compilation of a pool of items from which emergent factors are identified, the AQoL measures employ a conceptual approach to scale construction, with the aim of increasing the breadth of and sensitivity to a specified range of health features and states. In this approach, the target domains of QoL to be assessed are identified and relevant items are developed in consultation with panels of stakeholders, including researchers, clinicians and other health care professionals. Items are then refined within domains to identify items best characterising the health states of interest.

The Assessment of Quality of Life – 6D scale (AQoL-6D) is a relatively new addition to the AQoL family (previously labelled as the AQoL-II) and was developed in part to improve the content validity of QoL measures [3] and thus their sensitivity to a range of factors influencing life quality. In addition, the AQoL-6D aimed to create a measure appropriate for population health assessment, with increased sensitivity to wellness states compared to previous versions. The AQoL-6D is a 20 item assessment of six domains of QoL, characterised as ‘independent living’, ‘relationships’, ‘mental health’, ‘coping’, ‘pain’ and ‘senses’ and takes only a few minutes to complete. While the authors of the scale note that items assess both physical and psycho-social areas of QoL, these six domains may be combined to form a single global QoL factor.

In the process of constructing the AQoL-6D, additional items were developed for the AQoL item bank to increase coverage of QoL concepts both between and within domains [3]. Best performing items in terms of factor coherence and psychometric performance were determined from a construction sample, in which the overall factor structure was then confirmed [2-4]. As intended, the final 20 items formed a model with 3–4 items loading on each of the six domain scores, which in turn loaded onto a global QoL factor [2-4]. The AQoL-6D has been reported to discriminate between older persons at differential risks for falls [5], and to be associated with body mass index in adolescents, identifying the coping domain as particularly decreased in obese teens [6]. While these results suggest the AQoL-6D is a usable and sensitive instrument for a range of age groups, to date, there has been no published confirmation of the factor structure of the AQoL-6D outside of its original construction sample.

In light of its design for use in population health surveys and representation of physical and psycho-social aspects of QoL, the AQoL-6D was administered at the baseline and follow-up phases of two independently conceived but concurrent longitudinal community cohort studies conducted in New South Wales, Australia: 1) the Australian Rural Mental Health Study (ARMHS) [7], a project examining regional to very remote communities, which conducted baseline surveys in 2007–2009 and follow-up surveys from 2011–2012; and 2) the Hunter Community Study (HCS) [8], a project examining urban and inner regional communities around the Hunter Region, which conducted baseline surveys in 2004–2007 and follow-up surveys from 2010–2011. Under the auspices of the Extending Treatments, Education and Networks in Depression (xTEND) project [9], these cohorts have been combined with an aim to investigate issues of common interest and to maximise the utility of existing community surveys and national datasets. However, the comparability of the quality of life constructs assessed by the AQoL-6D across these cohorts, which encompass different age ranges and environmental contexts, is as yet unknown. Furthermore, concerns regarding ‘response shift bias’ are a potential problem in longitudinal studies of QoL [10], that is, when the conceptualization of an experience or state of being changes over time or with health states, resulting in confounding. Thus, confirming the structure and validity of the AQoL-6D scale across groups and timepoints is of importance not only for confirming its factor structure, but to facilitate meaningful and interpretable comparisons using this measure.

Using this large, aggregate longitudinal sample compiled for the purposes of the xTEND study, the current paper aims to: 1) review the performance of the AQoL-6D items and assess the internal validity and stability of the six associated domains; 2) confirm the overall factor structure of the AQoL-6D and its stability over time and across cohorts; 3) assess the concurrent validity of the AQoL-6D against an established measure of quality of life (the SF-36); and 4) assess the convergent validity of AQoL-6D factors by examining their association with indices of personal functioning (e.g., mental health functioning, psychological distress, satisfaction with life, physical functioning, body mass index, spirometry, pedometry and mobility).

## Methods

### Participants

Self-report postal survey data from two population-based cohort studies were combined to undertake the current study: the Hunter Community study (HCS) [8]; and the Australian Rural Mental Health Study (ARMHS) [7]. Detailed descriptions of recruitment, sample characteristics and methods employed can be obtained from their respective baseline descriptive papers. Briefly, the HCS is a study of persons aged 55–85 years residing in Newcastle, Australia, which was designed to assess a range of bio-psychosocial aspects of ageing. The ARMHS project includes persons aged 18 years and older residing in non-metropolitan areas, which was designed to assess mental health and wellbeing in rural and remote regions by over-sampling from remote and very remote populations. Both the HCS and ARMHS randomly selected potential participants from the state electoral roll. Introduction and recruitment letters were sent to individuals by post and non-responding individuals were followed-up by telephone calls. Overall, baseline response rates of 44.5% and 27.3% for the HCS (N = 3318) and ARMHS (N = 2639) respectively were achieved, with both samples having comparable rates of un-contactable or excluded persons (HCS: 26.9% and ARMHS: 25.2%). To reduce participant burden, survey items were administered over two baseline postal surveys in both cohorts: among respondents, 81.4% from the ARMHS and 97.4% from the HCS returned both surveys and were included in the current analyses. Between 2010 and 2012, 59.0% of baseline participants responded to a follow-up survey. Following ethical approval (Human Research Ethics Committees from the University of Newcastle and Hunter New England Area Health) individual participant survey data from the HCS and ARMHS were combined.

Table 1 displays information regarding numbers of cohort participants observed at baseline and follow-up phases, as well as the rationale for the selection of cases used in the current analyses. Of the combined N = 8896 baseline and follow up cases, 89.1% responded to all administered AQoL-6D items. Due to an administrative error, influenced by the perceived redundancy within the mental health items included in the survey, the mental health domain of the AQoL-6D (comprising 4 items) was not assessed in the ARMHS cohort at baseline. To address this issue, a subsample comprised of participants who had no more than 25% missing data on imputation model variables was used in the imputation of missing mental health domain items. This level of missing data has been demonstrated to produce minimal bias when using full information maximum likelihood estimation [11]. Following this procedure, the capacity of the multiple and single imputation sets of the mental health subscale to maintain the associative properties of the observed values (follow-up ARMHS, baseline and follow-up HCS) was examined. It was determined that a single imputation of mental health scale items for baseline ARMHS participants provided adequate representation of the missing values and these values were merged with the original dataset. A detailed account of the imputation and related analyses are provided in Additional file 1, which includes information regarding item response rates and floor/ceiling effects (see Additional file 1: Table S2). Participants with complete AQoL-6D data in the resulting overall set were used to assess the scale characteristics, structure, invariance and validity of the AQoL-6D (N = 7915).

**Table 1:** Description of number, origin and criteria for cases included in current analyses

	**Baseline**		**Follow up**		**Cohort N (group invariance)**		**Overall**
	**ARMHS**	**HCS**	**ARMHS**	**HCS**	**ARMHS**	**HCS**	
Returned both surveys	2149	3234	1261^#^	2252^#^			8896
Included in imputation procedure (<25% missing data on imputation model variables)	2127	3168	1234	2171			8700
Complete AQoL data^§^ (cases used for all reported analyses)	1987	2884	1111	1933	3098	4817	7915
**Phase N (temporal invariance)**	4871		3044				

^#^ Only one survey booklet was administered at follow-up, these values refer to N participants returning a follow-up survey. ^§^ Imputed values for the four mental health subscale items were merged into the original (un-imputed) set for ARMHS baseline participants.

### Measures

#### Quality of life

The Assessment of Quality of Life – 6D scale (AQoL-6D) is a 20-item self-report measure of QoL and general functioning [3]. Response options for each item include 4–6 levels, with higher scores indicating quality of life impairment. The 20 items of the AQoL-6D represent six domains, characterised as independent living (4 items), relationships (3 items), mental health (4 items), coping (3 items), pain (3 items) and senses (3 items). Items and response options are available online (http://www.aqol.com.au/) and reproduced in Additional file 2. The AQoL-6D was administered to the ARMHS and HCS cohorts at both baseline and follow-up phases.

Concurrent measurement of health related quality of life was conducted using the SF-36v1 (Australian version) [12], which was administered to HCS participants at both baseline and follow-up phases. The SF-36 is a well validated assessment of physical and mental health outcomes and has eight scales (physical functioning, role physical, social functioning, mental health, role emotional, vitality, bodily pain and general health) [13]. Scale scores were calculated according to the SF-36 manual [14], with items within scales recoded where necessary, summed and transformed to provide a scale score (range 0–100), with higher scores indicating greater health within that domain. The physical functioning and mental health scales of the SF-36 have been identified as ‘pure’ measures of their underlying constructs (physical and mental health respectively), with variability in each scale largely attributable to variation in its target health state [13]; for the current paper, these scales were used as concurrent indices, against which the AQoL-6D factors could be evaluated.

#### Other psychological functioning indicators

##### Psychological distress

The Kessler 10 (K10) [15] was used to assess current psychological distress and was administered to the ARMHS and HCS cohorts at both baseline and follow-up phases. The K10 is a 10-item self-report questionnaire that assesses the frequency of psychological distress over the past four weeks using a 5-point Likert scale: ‘none of the time’, ‘a little of the time’, ‘some of the time’, ‘most of the time’, ‘all of the time’. K10 scores range from 10 to 50, with higher scores denoting greater psychological distress. This measure displays good internal reliability (α = .93) and validity as a measure of psychological distress in community samples [16]. K10 items are also highly similar to AQoL-6D mental health subscale items, asking participants to rate the frequency with which they recently experienced anxious/negative affective states.

##### Life satisfaction

The Satisfaction With Life (SWL) scale was used to assess life satisfaction and was administered to the ARMHS cohort at baseline and to both the ARMHS and HCS cohorts at follow-up. The SWL [17] is a five-item scale measuring global life satisfaction, with participants rating each statement on a 7-point Likert scale (‘strongly disagree’ to ‘strongly agree’). A total global life satisfaction score is derived by summing all five items, with higher scores indicating greater life satisfaction (range = 5–35). The SWL is a widely used and well validated measure of life satisfaction [18] that displays good internal reliability (α = .87) [17].

#### Other physical functioning indicators

*Body mass index (BMI)*. At baseline, height and weight measurements were undertaken as part of a battery of clinical measures recorded by staff in the HCS, while the ARMHS obtained these measurements through self-reported survey responses. To address the significant potential for bias in the self-reporting of height and weight measurements, correction equations based on 2007–2008 Australian national survey data [16] were applied to self-reported height and weight indices for ARMHS participants before BMI was calculated. BMI was calculated as weight in kilograms divided by height in metres squared (kg/m^2^). BMI profiles by age for the sample are comparable to Australian population estimates compiled by the Australian Bureau of Statistics (see supplementary information provided in Additional file 1 for further details regarding the transformation and BMI profiles of the current sample).

Several other pertinent measures of physical functioning were collected in the HCS at baseline: *Pedometry* - a pedometer worn for 7 consecutive days during waking hours to record step count, from which mean daily steps was calculated; *Timed up and go* (TUG) - a measure of functional mobility that is operationalized as the time (in seconds) that a person takes to rise from a chair, walk three metres, turn around, walk back to the chair and sit down [19]; and *forced expiratory volume* (FEV) in 1 second - assessed (in litres) using electronic spirometers, together with Spida 5 software [20,21].

### Data analysis

Analyses were conducted using IBM SPSS Statistics for Windows v20.0 [22] and AMOS v20.0 [23]. Chi square tests were used for between group comparisons of categorical variables and one-way ANOVA for continuous variables. Unless otherwise stated, *p* < .01 was used as the threshold for all tests of statistical significance as a partial control for the number of statistical comparisons and the large number of observations.

#### Factor analyses

Maximum likelihood estimations were used for all confirmatory factor analyses (CFA). Correlation matrices used to produce all models reported here are provided in the supplementary documentation (Additional file 1: Tables S6). To assess the internal consistency of AQoL-6D domains, one factor congeneric models of each domain were constructed and the association of indicators with the domain, item variance explained by the domain (squared multiple correlation: SMC), and the reliability of scale items were inspected. Item reliabilities were assessed using both Cronbach’s alpha (α), which uses item correlations to assess a common construct, and Coefficient H [24], which uses model parameters to determine the reliability with which items assess a latent construct. As the majority of domains were just-identified [25] (reflecting the small number of items per domain), no fit statistics were calculated for these models.

To confirm that the six domains assessed a common underlying QoL construct, a one factor model of these domains was initially evaluated using CFA. Bagozzi et al.’s [26] method of determining discriminant validity, which utilizes nested models to assess whether scales are best represented by one or two higher-order factors, was used to assess the fit of competing models. The fit of a two factor solution, where the factor covariance was freely estimated (two correlated factors), was compared with a model where the correlation was constrained to be 1.00 (a single factor) and a chi-squared difference test conducted. To confirm whether the AQoL-6D factor structure was equivalent across the ARMHS and HCS samples and over time, multi-group confirmatory factor analyses (MGCFA) were conducted. Nested models were used to assess increasingly restrictive models of invariance across groups and time, to confirm that AQoL-6D factors displayed configural (indicators load on the same latent factor), metric (indicators contribute consistently to the latent factor), and variance/covariance invariance (the latent factor represents the same range of values and displays consistent relationships) across groups/time. Such multi-group methods present a widely accepted and powerful approach for testing measurement invariance [27]. Where models displayed group and temporal invariance, model parameters are reported for the overall sample. Model fit was assessed by inspecting absolute [standardised root mean square residual (SRMR) and root mean square error of approximation (RMSEA)] and incremental fit statistics [Tucker-Lewis Index (TLI), Comparative Fit Index (CFI)], as well as parameter estimates. Acceptable fit is indicated by RMSEA close to or less than .06, SRMR < .06 and incremental fit indices > .95 [28]. Where model fit was unacceptable, modification indices were inspected and considered in conjunction with the theoretical underpinnings of factors to improve model fit.

#### Psychometric analyses

Construction of aggregate AQoL-6D domain and factor scores for the sample are described and the stability of scores over-time assessed using the intra-class correlation coefficient (absolute) [29]. One-way ANOVAs were conducted to assess the influence of age and gender on factor and total scores and post-hoc age category comparisons using orthogonal polynomials were used to assess patterns of differences between age categories. The concurrent validity of AQoL-6D domain and factor scores was assessed against SF-36 domain scores. The magnitude of the associations between quality of life domains were examined using canonical correlations and Pearson’s correlation coefficient. Where multiple observations of an individual over time were available, analyses weighted and unweighted for the number of observations were conducted; correlation matrices for these analyses did not differ (maximum coefficient difference of r = .02) and unweighted analyses are reported in the manuscript. Factor score stability over time was contrasted with those of the SF-36 physical and mental health scales. The sensitivity of the SF-36 total score to impairment on each AQoL-6D domain was examined by standardizing AQoL-6D scores and plotting a score profile for those participants ranking in the lowest 25th percentile on the SF-36. We also build upon observations regarding the convergent validity of the AQoL-6D by examining the multiple correlation of AQoL-6D factor scores with sets of physical and psychological functioning indices (R^2^ with set) to assess the proportion of variance shared with these conceptually related constructs.

## Results

### Sample characteristics

Baseline and follow-up characteristics of the sample are presented in Table 2. Of the N = 2740 participants who provided AQoL-6D data at both timepoints, there was an average lag of 3.90 years (SD = 1.30) between baseline and follow-up surveys. Cohorts differed significantly in demographic and bio-psychosocial indices and cohort differences were largely consistent at both timepoints. Compared to baseline, follow-up participants were older, more likely to be married/de facto (ARMHS), had a higher level of education, were more likely to be retired (HCS), had lower psychological distress, and lower life satisfaction (ARMHS).

**Table 2:** Sample characteristics and comparisons by cohort and phase

	**Baseline – cohort comparisons**				**Follow up – cohort comparisons**				**Phase comparisons**		
	**HCS**	**ARMHS**	**Overall**	**p**	**HCS**	**ARMHS**	**Overall**	**p**	**HCS**	**ARMHS**	**Overall**
									**p**	**p**	**p**
Sample N	2884	1987	4871		1933	1111	3044				
**Socio demographic characteristics**											
Age (SD)	65.83 (7.59)	55.91 (4.03)	61.77 (11.76)	**	69.19 (7.13)	58.35 (12.9)	65.24 (10.96)	**	**	**	**
Female %	51.9	60.3	55.3	**	51.2	61.0	54.8	**	ns	ns	ns
Married/defacto %	75.3	71.2	73.6	**	75.4	78.6	76.6	ns	ns	**	*
High school complete %	77.8	71.3	75.2	**	81.3	76.3	79.5	**	*	*	**
Retired %	61.8	36.1	37.8	**	76.9	36.0	62.2	**	**	ns	**
**Indices of personal functioning (mean, SD)**											
Psychological distress (K10)	14.41 (5.31)	14.62 (5.16)	14.5 (5.25)	ns	13.67 (4.85)	13.48 (4.44)	13.6 (4.70)	ns	**	**	**
Life satisfaction (SWL)	.	25.82 (6.44)	.	.	25.51 (6.08)	24.74 (6.30)	25.22 (6.18)	**	.	**	**
Body Mass Index	28.70 (4.85)	28.07 (5.45)	28.43 (5.12)	**	.	.	.	.	.	.	.
Mental Health (SF-36)	79.72 (15.73)	.	.	.	79.84 (15.53)	.	.	.	ns	.	ns
Physical Functioning (SF-36)	73.31 (24.15)	.	.	.	74.07 (24.14)	.	.	.	ns	.	ns
FEV (litres)	2.43 (0.70)	.	.	.	.	.	.	.	.	.	.
TUG (seconds)	9.33 (2.71)	.	.	.	.	.	.	.	.	.	.
Steps per day (1000’s)	6.87 (3.18)	.	.	.	.	.	.	.	.	.	.

Note. * p < .01; ** p < .001; physical and psychological health indices did not deviate remarkably from available age relevant norms for these measures: K10 [30]; SWL [18]; BMI [31]; SF-36 [32]; forced expiratory volume (FEV) [33]; and timed up and go (TUG) [34,35]; while HCS step counts are similar to those of older persons in Switzerland, though greater than those in Colorado [36].

### Internal validity and structure

#### Items and domains

As detailed in Table 3, one factor congeneric modelling indicated that the AQoL-6D domains display positive associations with all of their component items and explained a considerable amount of item variance (SMC). Internal consistency of most domains was acceptable (Cronbach’s α range .73-.86), with the possible exception of the relationships (α = .63) and senses (α = .50) domains. The relationships domain had acceptable consistency in reference to Coefficient H (H = .76), which does not assume all items are equally good indicators of the latent construct, however, the consistency of the senses domain was still low (H = .61). Latent domains were generally a good fit to items, with the majority displaying high item reliabilities (SMC > .50), though relationships (aq5), senses (aq18 and aq20), and coping (aq12) domains contained some items with unacceptable reliability (SMC < .30).

**Table 3:** Raw and standardized coefficients from separate CFAs for each AQoL-6D domain (N = 7915)

**Domain**		**Item**		**B**	**SE**	**β**	**SMC**		**Error**	**Domain**	**Domain**
									**variance**	**α**	**H**
Independent living	-->	aq1	Household tasks	1.00		0.75	0.56	<-	0.30	0.86	0.88
Independent living	-->	aq2	Getting around	1.34	0.02	0.88	0.77	<-	0.21		
Independent living	-->	aq3	Walking	1.16	0.02	0.75	0.57	<-	0.39		
Independent living	-->	aq4	Self-care	0.71	0.01	0.73	0.54	<-	0.17		
Relationships	-->	aq5	Intimate relationships	1.00		0.32	0.10	<-	0.52	0.63	0.76
Relationships	-->	aq6	Health & family	1.53	0.07	0.81	0.66	<-	0.07		
Relationships	-->	aq7	Health & community	1.90	0.08	0.74	0.54	<-	0.17		
Mental health	-->	aq8	Despair	1.00		0.74	0.55	<-	0.25	0.79	0.80
Mental health	-->	aq9	Worry	1.11	0.02	0.75	0.56	<-	0.29		
Mental health	-->	aq10	Sadness	0.94	0.02	0.72	0.52	<-	0.25		
Mental health	-->	aq11	Agitation	0.63	0.01	0.59	0.34	<-	0.24		
Coping	-->	aq12	Energy	1.00		0.52	0.27	<-	0.44	0.73	0.77
Coping	-->	aq13	Control	1.35	0.04	0.79	0.63	<-	0.18		
Coping	-->	aq14	Coping	1.07	0.03	0.76	0.58	<-	0.13		
Pain	-->	aq15	Pain frequency	1.00		0.78	0.60	<-	0.39	0.84	0.85
Pain	-->	aq16	Pain severity	0.59	0.01	0.77	0.59	<-	0.14		
Pain	-->	aq17	Pain impact	1.06	0.02	0.85	0.72	<-	0.26		
Senses	-->	aq18	Vision	1.00		0.35	0.12	<-	0.37	0.50	0.61
Senses	-->	aq19	Hearing	2.58	0.19	0.73	0.54	<-	0.29		
Senses	-->	aq20	Communication	0.84	0.04	0.45	0.20	<-	0.15		

Note: *CFA* confirmatory factor analysis, *SMC* squared multiple correlation, *Domain α* Cronbach’s alpha coefficient for items within the domain, *Domain H* H coefficient for items within the domain.

#### Overall factor structure

Domain scores were initially calculated as the mean of the standardized factor weighted item scores, to both account for differing item reliabilities and reduce the inconsistency due to differing numbers of items and response options across domains. Standardized weights applied to item scores in the calculation of domain scores are presented in the supplementary documentation (Additional file 1: Table S5). CFA indicated a one-factor model of QoL displayed a positive association with all domains (β = .33-.81) and explained a reasonable amount of the variance in domain scores (SMC = .11-.65). However, model fit was poor [RMSEA = .20 (.19, .20), SRMR = .08; TLI = .70, CFI = .82], suggesting that the domain scores tapped somewhat dissimilar underlying constructs. Modification indices indicated that allowing the relationship between the mental health and coping domain errors to vary would reduce the discrepancy between the observed and optimal covariance matrixes by *χ*^2^ = 2122.37. This suggested that a two factor solution with mental health and coping representing a separate factor, perhaps assessing the psychological, as opposed to the physical, aspects of QoL, may improve the model. Model fit for the two factor solution was good [RMSEA = .07 (.07, .08), SRMR = .03; TLI = .96, CFI = .98]. A chi-square difference test indicated that the single factor solution significantly worsened the model compared with the two factor solution (*χ*^2^(1) = 2429.40, *p* < .001), indicating that the two constructs are reasonably different.

**Figure 1:**
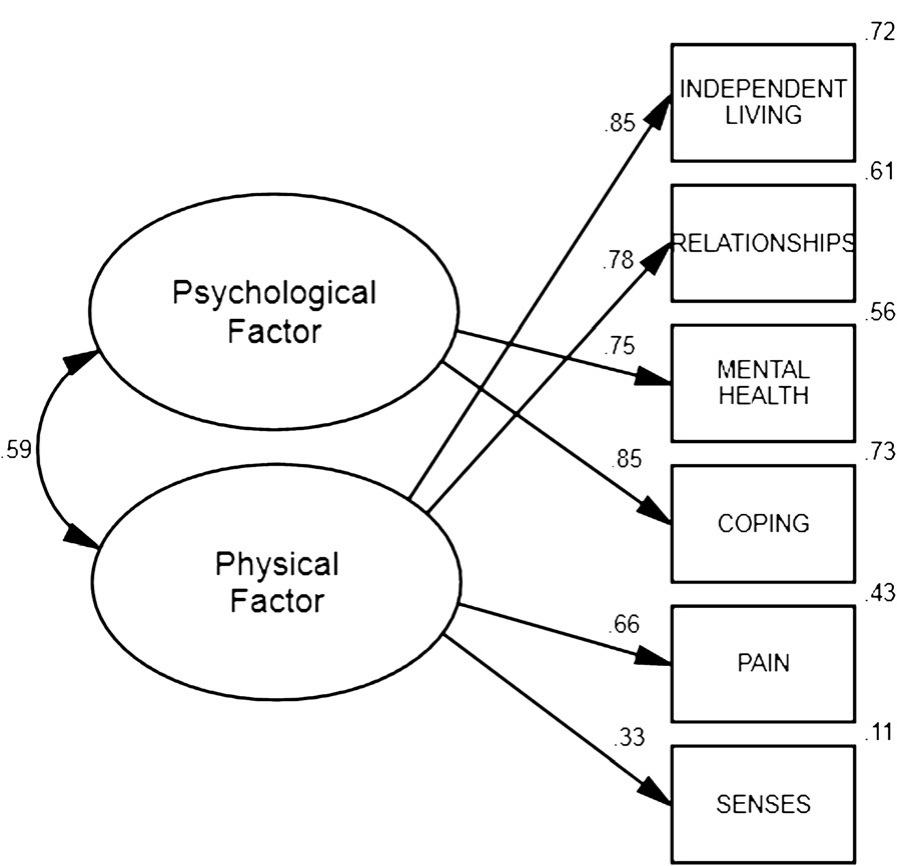
Standardized parameter estimates and squared multiple correlations for the two correlated higher-order factor structure of the six AQoL-6D domains (N = 7915).

Multi-group CFAs were conducted to assess whether this two factor solution was consistent across groups (ARMHS *vs.* HCS) and timepoints (baseline *vs.* follow-up). Results provided evidence that the two factor solution displayed configural [RMSEA = .06 (.05, .06), SRMR = .03; TLI = .95, CFI = .97], metric [RMSEA = .05 (.05, .06), SRMR = .03; TLI = .96, CFI = .97], and covariance/variance [RMSEA = .05 (.05, .06), SRMR = .04; TLI = .96, CFI = .97] invariance in both cohorts, suggesting this model was viable in both samples. Likewise, assessment of temporal invariance provided evidence that scales displayed configural [RMSEA = .06 (.05, .06), SRMR = .03; TLI = .95, CFI = .97], metric [RMSEA = .05 (.05, .05), SRMR = .03; TLI = .96, CFI = .97], and covariance/variance [RMSEA = .05 (.04, .05), SRMR = .04; TLI = .96, CFI = .97] invariance over time, suggesting this model was viable at both baseline and follow-up timepoints. Parameters for the two factor model are presented in Table 4 and its structure depicted in Figure 1.

**Table 4:** Model parameter estimates for two higher-order factors from a factor analysis of AQoL-6D domains (N = 7915)

**Factor**		**Domain**	**B**	**SE**	**β**	**SMC**		**Error variance**
Physical	-->	INDEPENDENT LIVING	0.61	0.01	0.85	0.72	<-	0.14
Physical	-->	RELATIONSHIPS	0.35	0.01	0.78	0.61	<-	0.08
Psychological	-->	MENTAL HEALTH	0.44	0.01	0.75	0.56	<-	0.15
Psychological	-->	COPING	0.46	0.01	0.85	0.73	<-	0.08
Physical	-->	PAIN	0.47	0.01	0.66	0.43	<-	0.29
Physical	-->	SENSES	0.17	0.01	0.33	0.11	<-	0.23
Physical	<-->	Psychological			0.59			

Note: *SMC* squared multiple correlation.

### Calculation of domain and factor scores

#### Domains: factor weighted scores *vs.* item weighted scores *vs.* utility scores

Correlations between standardized factor weighted domain scores and those derived by calculating the mean unit weighted domain score (i.e., giving equal weight to each item) were highly correlated for all scales [independent living r = .993; relationships r = .883; mental health r = .998; coping r = .962; pain: r = .996; senses r = .954]. The comparatively lower correlation for the relationships domain is due to the smaller standardized factor weighting of item aq5 (w = .055), which addresses general satisfaction with relationships, as opposed to items aq6 (w = .634) and aq7 (w = .311), which address the impact of an individual’s health on their ability to fulfil relationship roles (see Additional file 1: Table S5). Utility weighted domain scores [37] also correlated highly with mean unit weighted domain scores (range r = -.918 to r = -.983). Therefore, for parsimony, mean unit weighted domain scores were used for subsequent analysis and reporting (i.e., giving equal weight to items in calculating domain scores, and equal weight to domains in calculating factor scores).

#### Factor scores

AQoL-6D physical and psychological factors were calculated as the mean of their component unit weighted domain scores; both displayed a correlation of *r* = .99, *p* < .001 with their counterparts calculated as the mean of factor weighted domain scores. Internal consistency estimates, based on the set of items associated with each factor were, respectively, α = .86 and α = .84 for the physical and psychological factors. Physical and psychological factor scores displayed a correlation of *r* = .56, *p* < .001, which was highly consistent with the association observed in the two factor CFA model (see Figure 1). A total score, the mean of the physical and psychological factors, was also calculated. The internal consistency for the full set of AQoL-6D items contributing to the total score was α = .89. As expected (given their mathematical relationship), correlations between factors and the total score were high: physical *r* = .87, *p* < .001; psychological *r* = .90, *p* < .001).

Table 5 presents means and standard deviations for the domain and factor scores by gender and age, and provides a provisional set of norms for the proposed AQoL-6D scoring scheme. There was a significant but small effect of gender on both physical (F(1, 7915) = 34.21, *p* < .001) and psychological (F(1, 7914) = 51.34, *p* < .001) factor scores, with men reporting greater impairment on physical and women reporting greater impairment on psychological factors. There was no influence of gender on the total score (F(1, 7914) = 1.35, *p* = .246). There was no significant influence of age category on psychological functioning (F(4, 7901) = 3.06, *p* = .016) but a significant effect of age category on physical functioning (F(4, 7901) = 138.21, *p* < .001) and the total score (F(4, 7901) = 33.082, *p* < .001). Post-hoc age category comparisons revealed significant linear components of trend for both the physical functioning (F(1, 7897) = 551.13, p < .001) and total scores (F(1, 7897) = 132.82, p < .001), indicating a progressive increase in impairment with increased age. No higher order (i.e., non-linear) effects were observed for any of these scores.

**Table 5:** Mean (SD) AQoL-6D quality of life impairment domain and factor scores by gender and age

**Group**	**Age (years)**	**N**	**Independent living**	**Relationships**	**Mental health**	**Coping**	**Pain**	**Senses**	**Psychological factor**	**Physical factor**	**TOTAL**
Male	18-34	53	1.42 (0.64)	1.31 (0.36)	1.92 (0.62)	1.81 (0.67)	1.55 (0.74)	1.40 (0.42)	1.87 (0.59)	1.42 (0.40)	1.64 (0.44)
	35-44	110	1.41 (0.50)	1.29 (0.42)	1.95 (0.59)	2.01 (0.59)	1.68 (0.62)	1.75 (0.46)	1.98 (0.54)	1.53 (0.40)	1.75 (0.43)
	45-54	265	1.45 (0.52)	1.36 (0.46)	1.85 (0.64)	1.99 (0.60)	1.73 (0.67)	1.87 (0.47)	1.92 (0.57)	1.60 (0.38)	1.76 (0.42)
	55-64	1343	1.63 (0.64)	1.38 (0.44)	1.90 (0.59)	2.00 (0.57)	1.84 (0.73)	2.01 (0.44)	1.95 (0.53)	1.72 (0.43)	1.83 (0.43)
	65+	1772	1.93 (0.74)	1.48 (0.52)	1.83 (0.54)	2.01 (0.53)	1.86 (0.74)	2.07 (0.45)	1.92 (0.49)	1.84 (0.47)	1.88 (0.43)
	Total	3543	1.76 (0.70)	1.43 (0.48)	1.86 (0.57)	2.00 (0.56)	1.83 (0.73)	2.01 (0.46)	1.93 (0.51)	1.76 (0.45)	1.85 (0.43)
Female	18-34	149	1.34 (0.41)	1.25 (0.34)	1.93 (0.57)	1.98 (0.61)	1.34 (0.54)	1.41 (0.37)	1.95 (0.54)	1.33 (0.30)	1.64 (0.37)
	35-44	275	1.37 (0.49)	1.32 (0.40)	1.99 (0.63)	2.12 (0.59)	1.50 (0.63)	1.56 (0.42)	2.06 (0.56)	1.44 (0.36)	1.75 (0.42)
	45-54	414	1.46 (0.51)	1.33 (0.40)	1.91 (0.54)	2.06 (0.52)	1.73 (0.67)	1.82 (0.38)	1.99 (0.48)	1.58 (0.36)	1.79 (0.38)
	55-64	1648	1.62 (0.61)	1.38 (0.40)	1.99 (0.57)	2.07 (0.52)	1.87 (0.74)	1.83 (0.39)	2.03 (0.49)	1.67 (0.41)	1.85 (0.40)
	65+	1873	1.96 (0.77)	1.48 (0.47)	1.94 (0.56)	2.07 (0.53)	1.93 (0.76)	1.89 (0.42)	2.01 (0.49)	1.81 (0.47)	1.91 (0.42)
	Total	4359	1.73 (0.70)	1.41 (0.44)	1.96 (0.57)	2.07 (0.53)	1.84 (0.74)	1.82 (0.42)	2.02 (0.49)	1.70 (0.44)	1.86 (0.41)
Overall	18-34	202	1.36 (0.48)	1.27 (0.35)	1.93 (0.58)	1.94 (0.63)	1.39 (0.6)	1.41 (0.38)	1.93 (0.56)	1.36 (0.33)	1.64 (0.39)
	35-44	385	1.38 (0.49)	1.31 (0.40)	1.98 (0.62)	2.09 (0.59)	1.55 (0.63)	1.61 (0.44)	2.04 (0.56)	1.47 (0.37)	1.75 (0.42)
	45-54	679	1.46 (0.52)	1.34 (0.42)	1.89 (0.58)	2.03 (0.55)	1.73 (0.67)	1.84 (0.41)	1.96 (0.52)	1.59 (0.37)	1.78 (0.39)
	55-64	2991	1.63 (0.63)	1.38 (0.42)	1.95 (0.58)	2.04 (0.54)	1.85 (0.73)	1.91 (0.42)	1.99 (0.51)	1.69 (0.42)	1.84 (0.41)
	65+	3645	1.94 (0.75)	1.48 (0.49)	1.89 (0.55)	2.04 (0.53)	1.89 (0.75)	1.98 (0.45)	1.97 (0.49)	1.82 (0.47)	1.90 (0.43)
	Total	7915	1.74 (0.70)	1.42 (0.46)	1.92 (0.57)	2.04 (0.54)	1.84 (0.73)	1.91 (0.45)	1.98 (0.50)	1.73 (0.45)	1.85 (0.42)
Temporal stability (*r*_icc_)		2740	0.70	0.57	0.55	0.63	0.67	0.62	0.65	0.75	0.73

Note: Age category for N = 5 women and N = 8 men from HCS were unknown; *r*_icc_ = intra-class correlation coefficient (absolute); *all p < .001*; for all measures, higher scores indicate a poorer quality of life.

### Concurrent validity

Associations between AQoL-6D domains and SF-36 scales are reported in Table 6. All domain scores displayed significant negative associations, indicating that increased impairment on AQoL-6D domains was associated with poorer quality of life on the SF-36 scales. Conceptually related scales were more highly related than those that were not conceptually related, supporting the convergent validity of the AQoL-6D domains. The canonical correlation (six AQoL-6D domains *vs.* eight SF-36 scales) revealed a high level of shared variance (R_c_ = .884 or 78% shared) between these sets, suggesting that the two sets measure highly similar aspects of life quality.

**Table 6:** Pearson’s correlation coefficients between AQoL-6D domains and SF-36 scales

**SF-36 Scales**	**N**	**AQoL-6D Domains**					
		**Independent living**	**Relationships**	**Mental health**	**Coping**	**Pain**	**Senses**
Physical functioning	4748	**-0.77**	-0.54	-0.29	-0.44	-0.58	-0.22
Role physical	4701	-0.59	-0.47	-0.30	-0.42	-0.51	-0.23
Social functioning	4795	-0.52	-0.52	-0.51	-0.51	-0.45	-0.24
Mental health	4758	-0.32	-0.42	**-0.72**	**-0.60**	-0.33	-0.23
Role emotional	4692	-0.38	-0.41	-0.46	-0.44	-0.31	-0.20
Vitality	4762	-0.56	-0.51	-0.53	**-0.68**	-0.49	-0.27
Bodily pain	4783	-0.57	-0.43	-0.33	-0.38	**-0.80**	-0.20
General health	4693	**-0.60**	-0.52	-0.39	-0.53	-0.53	-0.28

Note: All p < .001; correlations of 0.60 or higher are highlighted in bold font.

The stability of domain and factor scores over time are presented as intra-class correlation coefficients at the bottom of Table 5. All domain and factor scores displayed moderate stability over time (*r*_icc_ range .55-.75). Temporal stability of the AQoL-6D psychological factor was lower than that of the physical factor, a pattern that is consistent with that observed for the SF-36 mental health and physical functioning scales (see Table 7).

**Table 7:** Associations of AQoL-6D summary scores with concurrent assessments of quality of life (SF-36) and with independent indices of physical and psychological functioning

				**AQoL-6D summary scores**				
		**Physical factor**		**Psychological factor**		**Total score**		**Temporal stability**
	**N**	**r**	**R**^**2 **^**with set**	**r**	**R**^**2 **^**with set**	**r**	**R**^**2 **^**with set**	**r**_**ICC**_
**Physical indices**								
Physical functioning SF-36	4748	-0.73	.502	-0.40	.132	-0.63	.354	.65 (N = 1733)
Body mass index	4433	0.24		0.10		0.19		.
Timed up and go	2554	0.44		0.19		0.35		.
Pedometry	2217	-0.30		-0.10		-0.22		.
Forced expiratory volume	2312	-0.16		-0.09		-0.14		.
**Psychological indices**								
Mental health SF-36	4758	-0.43	.246	-0.74	.606	-0.66	.518	.56 (N = 1735)
Psychological distress	7831	0.44		0.71		0.66		.57 (N = 2692)
Life satisfaction	4899	-0.34		-0.55		-0.52		.64 (N = 951)

Note: All *p* < .001; pedometry = mean steps per day; r_ICC_ = intra-class correlation (absolute); R^2^ with set based on HCS baseline participants only: physical indices N = 1973, and psychological indices N = 1808.

**Figure 2:**
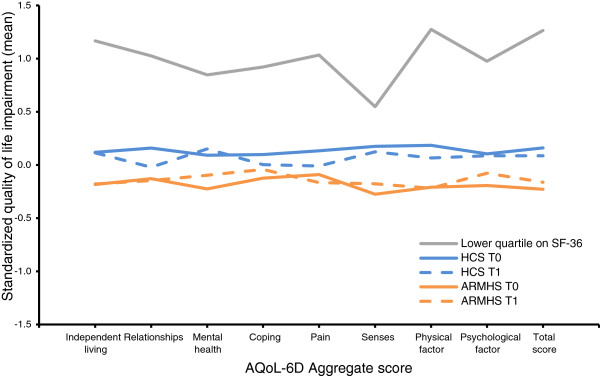
Profile plot of standardized AQoL-6D domain, factor and total scores by group and for those with poorest quality of life on the SF-36.

Figure 2 presents a profile plot of standardized AQoL-6D domain and factor scores for each cohort by phase. Compared to the HCS, ARMHS participants displayed lower impairment across all domains. The sub-group scoring below the 25th percentile on the SF-36 (i.e. lower than 63.50 on a mean total of the SF-36 scales) generally reported greater impairment (approximately 1 standard deviation above the mean) on all subscales of the AQoL-6D. However, impairment experienced in this subgroup on the AQoL-6D senses domain dropped to around half a standard deviation above the mean.

### Convergent validity

Results for the correlation and multiple correlation of factor and total scores with other indices of physical and psychological functioning are presented in Table 7. Greater impairment on AQoL-6D summary scores was associated with poorer functioning on all psychological and physical indices. The psychological factor displayed higher correlations with concurrent psychological indices, while the physical factor displayed higher correlations with the concurrent physical indices. Similarly, the psychological factor displayed a greater proportion of shared variance with psychological than physical functioning indices, while the physical factor displayed a greater proportion of shared variance with physical than psychological functioning indices.

## Discussion

The AQoL-6D was designed to be sensitive to a range of health domains and an increased spectrum of wellness-illness states, and to be suitable for administration in community cohorts. The current analyses build upon the recent work of Richardson et al. [3] reporting on the processes of ensuring the content validity of the AQoL-6D. Utilising individual participant data drawn from two large longitudinal community cohorts, the current study confirms that the instrument was acceptable to participants even when administered within an extensive survey battery, with 89% completing all administered AQoL-6D items. In line with previous work [3], questions regarding close and intimate relationships had the poorest response rate (3% missing). Current results largely confirm the internal consistency of AQoL-6D domains, providing evidence that the items within domains assess a single underlying construct. Comparison of nested CFA models suggested AQoL-6D domains are best represented by two correlated higher-order factors representing the physical and psychological aspects of life quality impairment. This model produced good fit across demographically diverse cohorts, as well as over time. Observations that increased age is associated with poorer physical quality of life, with little or no effect of age on psychological quality of life, are consistent with other Australian population surveys utilizing QoL instruments with multidimensional scoring [38-41].

### Domain structure and validity

Characterising the handicap associated with a representative range of experiences of impairment was an explicit goal in the development of the AQoL-6D. One-factor congeneric models and indices of internal consistency suggest the items provided a cohesive representation of their underlying domains, though the internal consistency of the relationships (α = .63) and senses (α = .50) domains was relatively poor. Items within the relationships domain address somewhat different aspects of social interactions (including general satisfaction with relationships, and the impact of an individual’s health on their ability to fulfil relationship roles). The senses domain was designed to assess the impairment associated with sensory dysfunction (visual, hearing and communication) and, while dysfunctions do not necessarily co-occur, they do pose similar barriers to social functioning and thus life quality. The reduction in consistency within these scales thus represents a trade-off between domain cohesion and sensitivity.

Analyses also support the concurrent validity of AQoL-6D domain scores against SF-36 scale scores. Domain scores constructed from mean item scores were highly consistent with those of the SF-36, with the two sets of scores having 78% shared variance. Scores for common constructs consistently demonstrated strong associations: independent living was highly associated with SF-36 physical functioning and most weakly associated with SF-36 mental health, with the opposite effect observed for AQoL-6D mental health; pain and SF-36 bodily pain were highly related, with weak associations observed with SF-36 mental health and role emotional scales; and coping displayed its strongest association with SF-36 vitality and weakest with bodily pain scale. Two AQoL-6D domains did not display such convergence with other SF-36 scales: relationships was moderately associated with all SF-36 scales, likely reflecting the component item’s predominant representation of the impact of physical functioning on relationships, rather than general social wellbeing or the impact of psychological functioning on relationships; and senses was weakly associated with all SF-36 scales, suggesting the absence of an analogous assessment of sensory impairment in the SF-36. This inference was also supported by our plot of standardized AQoL-6D domain scores for those with poorest quality of life on the SF-36 (see Figure 2). This plot demonstrated that while the remaining five AQoL-6D subscales for this group were at least one standard deviation above the mean, scores on the sensory domain were only half a standard deviation above the mean, reflecting an insensitivity of the SF-36 to experiences of sensory impairment as assessed by the AQoL-6D.

### Factor structure and validity

Current results show two correlated but divergent factors, here characterized as physical (independent living, relationships, pain and senses) and psychological (mental health and coping) factors, provide a better explanation of the model variance than a single global QoL factor in our community sample. The moderate correlation between scores on the physical and psychological factors (*r* = .59) demonstrates that they are not completely independent. Several global and disease specific scales assessing QoL support the existence, association and utility of these divergent factors (i.e., the SF-36 [13]). There are several possible reasons that current results regarding the fit of a single factor model for the AQoL-6D differed from those observed in its construction sample. During its construction, the final 20 AQoL-6D items were administered within a survey of 112 items to a sample of community members (N = 316), hospital outpatients (N = 206) and inpatients (N = 96). The completion of a large number of similar items may have reduced each participant’s capacity to discriminate between health states due to fatigue or contributed to the emergence of a particular response pattern. Further, approximately half of this relatively small sample was drawn from hospital services. Comorbidity of poor physical and psychological quality of life may be greater in such samples than in the general community, resulting in greater differentiation of aspects of life quality in community compared to hospital based samples. Research contrasting model fit for community and hospital samples may be necessary to determine the most appropriate factor structure for representing quality of life in such populations.

The availability of multiple groups and timepoints in which to confirm the AQoL-6D factor structure is a strength of the current work. While not often tested or acknowledged, when the same variable is compared across groups or timepoints it is assumed that the measure is interpretable as the same construct across observations. The current study formally addressed these assumptions using multi-group confirmatory factor analyses. These analyses place increasingly stringent constraints upon the likeness of the model across groups, to assess whether the theoretical model fitted the observed data in both groups. The current analyses assessed whether: (1) the domains were associated with the same latent factor (configural invariance); (2) the domains were associated with the latent factor with the same strength and direction (metric invariance); and (3) the latent factors represented the same range of values and were related to each other with the same strength (variance/co-variance invariance). The first two of these represent assessments of measurement invariance. The latter is a test of structural invariance (i.e., comparable value ranges and relationships across groups and times); while included largely for theoretical reasons, this analysis confirmed that the AQoL-6D factors provide consistent representations of overall life quality. Thus, the two factor model displayed a psychometrically and theoretically meaningful representation of life quality across two community groups which differ on a range of demographic, bio-psychosocial and contextual indices. The two-factor model was replicated over time, suggesting the two factor solution is also suitable for assessing performance over time. This demonstration of invariance facilitates confident interpretation and, for the xTEND project, encourages us to undertake future examinations of the cross-sectional and longitudinal drivers of QoL (e.g., the impacts of chronic illness, social factors such as retirement, and community remoteness).

The AQoL-6D factor scores displayed convergent validity in their associations with a range of other indices of physical and psychological functioning. Temporal stability of domains and factors over a three year follow-up period was consistent with the patterns observed for the SF-36. Physical factor scores were more strongly associated with measures of physical functioning, as assessed by the SF-36, BMI, mobility, pedometry and spirometry, and explained 50.2% of variability in this set of indices but only 24.6% in the psychological indices. Similarly, psychological factor scores were associated predominantly with psychological indices of mental health, psychological distress, and life satisfaction, and explained 60.6% of variability in this set of indices but only 13.2% in the physical indices. Such evidence provides support for the differential sensitivity of these factors to associated physical and psychological states.

Our analyses also confirmed that the proposed AQoL-6D total score displayed comparable associations with the SF-36 physical and mental health scales (see Table 7), suggesting that an aggregate based on either measure would tend to have similar properties. As with any other composite score, such an aggregation would also tend to underestimate associations with factors that were differentially linked to the underlying components; in the current study, for example, the physical and psychological aspects of quality of life were differentially associated with gender and age, associations that would be obscured if only total scores were used. On the other hand, such a summary score could be of interest to researchers wishing to obtain a global rating of QoL, from which to broadly characterise their sample and/or to track changes over time; based on the current analyses, for example, gender differences could be largely ignored if the AQoL-6D total score was the primary focus. In short, the research value of composite scores depends on the context and the questions of interest (*cf*., two factors based on psychometric scoring *vs.* a single utility index).

### Practical issues

As noted previously, utility weights have been developed for the AQoL-6D. Utility weighting is commonly used in an effort to increase the interpretability of quality of life scores as a trade-off between quantity and quality of life, by accounting for preferences for health states; however, caution is advised in their interpretation and population specific weights accounting for preferences for health states are required. While utility measures have been popular in the health-related decision making literature, there has been relatively low interest in health utilities in relation to mental health treatment decision making. In econometric QoL studies, the motivation for choosing a particular QoL measure may be to provide an index by which health related burden or cost can be estimated (e.g., quality adjusted life years or cost-utility measures); in which case, the multidimensional nature of QoL may not be important or useful, and a single index may be desirable [42]. However, if the motivation for instrument selection is to assess the determinants of wellbeing and their outcomes, as is often the case in the social sciences, acknowledging the multidimensional nature of QoL is of considerable importance and may have several psychometric benefits. For example, by acknowledging the divergent qualities of these factors, we may be able to produce QoL scores with greater external validity and sensitivity to a broader range of determinants and outcomes. Investigation of the burdens and determinants of physical and mental health outcomes present a situation in which QoL and its correlates are of greater interest. Using the simple scoring routine described in the current paper (and reproduced in Additional file 2), a set of preliminary age and gender normative scores were derived. While they could be improved through increased representation of persons under 55 years of age, particularly in urban areas, to our knowledge, these provisional norms provide the largest and most representative collection of AQoL-6D community data to date.

The current paper may also help inform researchers in the selection of instruments for administration in the general community. We present evidence that the AQoL-6D domains and factor scores depicted here display construct validity and are interpretable over a range of community contexts. Additionally, while the AQoL-6D displays a high level of commonality with a concurrent assessment of quality of life, the SF-36, there are several points of difference between these instruments, including: a smaller number of items (20 *vs.* 36) assessed in the AQoL-6D; the differing aspects of relationships measured; and the absence of an explicit assessment of the impact of sensory impairment on life quality in the SF-36 scale. The sensory domain could be particularly important in assessing health related life quality in older groups and for persons living in non-urban areas, where often fewer facilities are provided or adapted to assist persons experiencing sensory (e.g., visual impairment) or physical disability. Researchers planning to assess quality of life experienced in the community should consider the relative value of these measures for addressing their research questions.

### Limitations

A potential limitation of the current study lays in the imputation of missing baseline mental health item data. However, in this instance, the apparent cause of data missingness is known (the items were inadvertently omitted from the baseline ARMHS survey). This situation is similar to that of planned missingness designs [43,44], wherein random sections of a cohort are asked subsets of questions for purposes of maximising the amount of information derived while reducing survey length. Moreover, in the current study, the imputed data performed as expected with respect to item and domain profiles, comparisons across cohorts and phases, and relationships with other scales.

A second potential limitation relates to the exclusion of participants without complete data. As the purpose of this paper was to describe the structure, group and temporal invariance of the AQoL-6D across two large cohorts, and a relatively low proportion of participants had incomplete data (11%), it was judged that observations with complete data were adequate to characterise the variability observed across cohorts and phases.

A third limitation relates to current results regarding the concurrent and convergent validity of the AQoL-6D. These results are largely based on associations from an older sample of persons from urban-inner regional areas (i.e., the Hunter Community Study); for example, the reported associations of the SF-36 and physiological measurements with the AQoL-6D may differ from findings based on younger age groups.

Finally, there is ongoing debate regarding the appropriate statistics for reporting in CFA. While the majority of sources recommend that multiple fit indices should be considered in assessing model fit, some argue that the vulnerabilities of the *χ*2 statistic to large sample sizes may distract from reasonable model fit [45]. Others suggest that it is wrong to suggest that the non-perfect absolute model fit indicated by the *χ*2 statistic is necessarily trivial, and should provide a basis for investigating model misspecification [46]. The purpose of the current analyses was to identify a coherent structure under which an existing brief instrument characterised by coherent domain scores could be meaningfully aggregated; consequently, we have not reported the associated *χ*2 statistics, instead assessing the variance explained by the factor scores. In short, the parsimony and interpretability of the model was our primary goal – to model practical methods of characterising, scoring and interpreting the aggregate descriptive system of the AQoL-6D – in our case, for the ongoing purposes of the xTEND project, but we are happy to share and recommend this approach to the calculation of AQoL-6D summary scores.

## Conclusions

To our knowledge, this is the first study to assess the factor structure of the AQoL-6D outside its construction sample. We were able to confirm the internal validity of the six domains assessed by the AQoL-6D. These scores displayed a moderate level of temporal stability over the four year follow-up period, with physical factors displaying greater stability than psychological factors. Current findings suggest a two factor model, characterised here as physical and psychological quality of life impairment, provides the best fit for the data when the AQoL-6D is administered as described in the general community. This model fits equally as well over two diverse cohorts and over the four year follow-up period. The concurrent validity of domain and factor scores were upheld in light of their strong associations and shared variance with an established concurrent measure of health related quality of life. Evidence for the convergent validity of factors was demonstrated through a higher proportion of shared variance with corresponding domains of physical and psychological indices of personal functioning. The xTEND study demonstrates the value of pooling individual participant data from comparable longitudinal cohorts, particularly for the purposes of scale validation, where issues of factor invariance across groups and time are otherwise of concern, but untestable.

### Endnotes

^a^ For the purposes of this paper, the terms ‘items’, ‘domains and ‘factors’ will be used in a consistent manner. ‘Items’ refer to the individual question(s) to which participants respond, ‘domains’ refer to the first-order factors these items characterise, and ‘factors’ refer to the second-order factors to which these domains relate (i.e., items can be combined to form domains, and domains can be combined to form factors).

## Abbreviations

QoL: Quality of life; AQoL: Assessment of Quality of Life; SF-36: The Short Form (36) health survey; ARMHS: Australian Rural Mental Health Study; HCS: Hunter Community Study; xTEND: Extending Treatments, Education and Networks for Depression; K10: Kessler 10; BMI: Body mass index; TUG: Timed up and go; FEV: Forced expiratory volume; CFA: Confirmatory factor analyses; MGCFA: Multi-group confirmatory factor analyses; CFI: Comparative fit index; TLI: Tucker-Lewis index; RMSEA: Root mean square error of approximation; SRMR: Standardised root mean square residual; SMC: Squared multiple correlation.

## Competing interests

The authors declare that they have no competing interests.

## Authors’ contributions

BJK and TJL led the ARMHS study and JRA led the HCS study from 2010. BJK, KJI, JRA and TJL led the program of research associated with the combination of these studies as the eXtending Treatments, Education and Networks for Depression (xTEND) project. JA and TJL undertook the statistical modelling and generated the results. All authors provided interpretation of the results. JA drafted the manuscript and all authors contributed to its editing. All authors read and approved the final manuscript.

## Supplementary Material

Additional file 1 Supplementary documentation and data. Click here for file

Additional file 2 Appendix 1 AQoL-6D items and scoring. Click here for file
