# Supplementary material for: Construct validity of the Assessment of Quality of Life - 6D (AQoL-6D) in community samples
Source: Health Qual Life Outcomes. 2013 Apr 17;11:61. doi: 10.1186/1477-7525-11-61 (PMC3639231; doi:10.1186/1477-7525-11-61)
Supplement: Additional file 1 — Supplementary documentation and data. [file 1477-7525-11-61-S1.docx]

This document provides supplementary information for the paper by Allen et al. (2013) titled: “Construct validity of the Assessment of Quality of Life – 6D (AQoL-6D) in community samples”.

Contents

[BMI conversion: corrections for self-reported height and weight measurements 2](#_Toc350507105)

[Figure S1 Mean BMI by age calculated using self-reported, corrected and measured height and weight data for A) male participants, and B) female participants 2](#_Toc350507106)

[Comparison of BMI by age with normative data from the Australian Bureau of Statistics 3](#_Toc350507107)

[Data imputation 4](#_Toc350507108)

[Table S2 Missing value analyses and description of observed item values (N = 8,700) 5](#_Toc350507109)

[Table S3 Estimates for AQoL mental health domain items over 5 imputations 6](#_Toc350507110)

[Table S4 Comparison of stability of association (β) of a summed total of mental health items regressed onto selected predictors across pooled and single set estimates and with observed associations at HCS baseline and ARMHS and HCS follow-up. 7](#_Toc350507111)

[Table S5 Raw and standardized weights applied to items to created weighted domain scores 8](#_Toc350507112)

[Table S6 Correlation Matrixes from CFA and SEM analyses 9](#_Toc350507113)

[Subscale CFA 9](#_Toc350507114)

[Independent living 9](#_Toc350507115)

[Relationships 9](#_Toc350507116)

[Mental health 9](#_Toc350507117)

[Coping 9](#_Toc350507118)

[Pain 10](#_Toc350507119)

[Senses 10](#_Toc350507120)

[Overall CFA 11](#_Toc350507121)

[MGCFA: Cohort invariance 12](#_Toc350507122)

[ARMHS 12](#_Toc350507123)

[HCS 12](#_Toc350507124)

[MGCFA: Temporal invariance 13](#_Toc350507125)

[Baseline 13](#_Toc350507126)

[Follow-up 13](#_Toc350507127)

[Glossary 14](#_Toc350507128)

[Additional References 15](#_Toc350507129)

# BMI conversion: corrections for self-reported height and weight measurements

It is known that self-report measures of height and weight, as used in the ARMHS cohort, are subject to bias (over estimating height and under estimating weight), resulting in under estimation of BMI, though evidence suggests this bias is in decline in Australia and the US [[1](#_ENREF_1), [2](#_ENREF_2)]. To address the significant potential for bias in the self-reporting of height and weight measurements, correction equations based on 2007-2008 Australian national survey data [[1](#_ENREF_1)] were applied to self-reported height and weight indices for ARMHS participants, factoring in age and gender of respondent before BMI was calculated. For illustration, supplementary Figure S1 displays BMI by age and gender for all cohort participants aged over 55 (N = 4076) calculated from self-reported (ARMHS: N = 1180, Men = 518, Women = 662), adjusted self-reported (ARMHS) and measured (HCS: N = 2896, Men = 1399, Women = 1497) height and weight data. Figures show that while the self-reported height and weight measures resulted in consistently lower mean BMI values across age and gender groups, the application of correction equations produced mean BMI values for the ARMHS cohort that were closer to mean measured BMI values in the HCS cohort. The magnitude of the association between BMI and the AQoL-6D domains scores was highly consistent across cohorts: psychological (ARMHS: r(1878) = .092, p < .001; HCS: r(2555) = .092, p < .001) physical (ARMHS: r(1878) = .222, p < .001; HCS: r(2555) = .247, p < .001) and total (ARMHS: r(1878) = .173, p < .001; HCS: r(2555) = .189, p < .001). These observations provide evidence that application of correction equations to self-reported height and weight measurements significantly reduces biases in calculated BMI, and there was only modest evidence for such a bias in the current data sets.

| 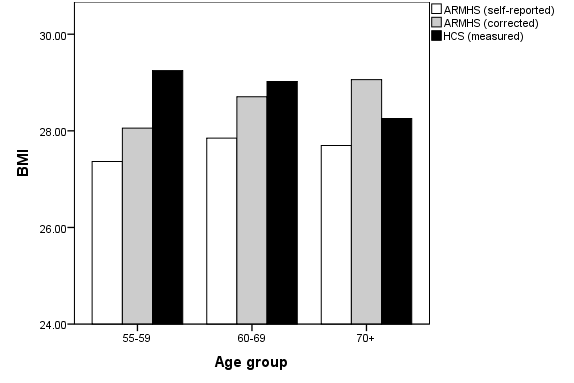  A | 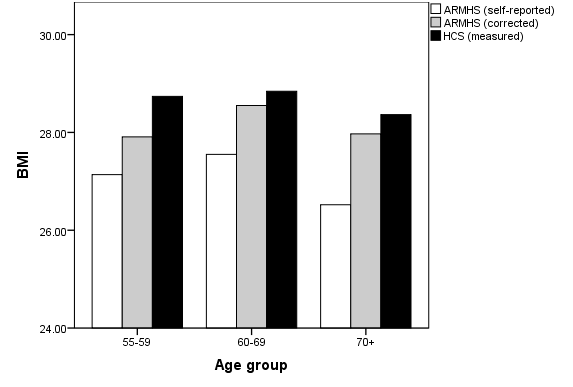  B |
| --- | --- |
| Figure S1 Mean BMI by age calculated using self-reported, corrected and measured height and weight data for A) male participants, and B) female participants | |

# Comparison of BMI by age with normative data from the Australian Bureau of Statistics

Data regarding population breakdown of BMI category membership by age from the Australian Bureau of Statistics [[3](#_ENREF_3)] alongside data from the xTEND study are presented in Table S1. Our estimates are broadly comparable with those of the population suggesting our sample were broadly representative of persons by weight in the Australian community.

Table S1 proportion of persons in BMI category by age group from the Australian Bureau of statistics (2012) and the xTEND study

|  | ABS data (2011-2011) | | | | |  |
| --- | --- | --- | --- | --- | --- | --- |
| **Persons** | **25–34** | **35–44** | **45–54** | **55–64** | **65–74** |  |
| Underweight (BMI less than 18.50) | 1.7 | *0.5 | *0.9 | *0.4 | *0.5 |  |
| Total Normal range (BMI 18.50–24.99) | 43.8 | 34.7 | 26.4 | 25.9 | 24.8 |  |
| Overweight (BMI 25.00–29.99) | 32.2 | 36.1 | 40.0 | 37.4 | 38.9 |  |
| Obese (BMI 30.00 or more) | 22.3 | 28.7 | 32.7 | 36.3 | 35.8 |  |
|  | xTEND data | | | | |  |
|  | ***18-34*** | **35–44** | **45–54** | **55–64** | ***65+*** |  |
| Underweight (BMI less than 18.50) | 2.1 | 0.4 | 0.5 | 0.4 | 0.7 |  |
| Total Normal range (BMI 18.50–24.99) | 48.9 | 36.4 | 32.4 | 21.5 | 20.4 |  |
| Overweight (BMI 25.00–29.99) | 28.4 | 37.2 | 39.3 | 45.4 | 46.3 |  |
| Obese (BMI 30.00 or more) | 20.6 | 25.9 | 27.8 | 32.7 | 32.6 |  |
| * estimate has a relative standard error of 25% to 50% and should be used with caution | | | | | | |

# Data imputation

As one of the goals of imputing the four missing AQoL-6D baseline mental health domain items in the ARMHS sample was to obtain baseline values that could be used in a consistent fashion in future analyses, an expectation-maximization approach to imputation [[4](#_ENREF_4)] was initially chosen, in which a single set of values was imputed from expectation-maximization parameters. However, inspection of resulting datasets indicated that the imputed values were biased in their relationship to other highly associated factors such as the K10, likely due to inadequate estimation of random error in this procedure. Consequently, to avoid distorting the associations between imputed values and variables commonly associated with indices of psychological distress, five datasets predicting these four missing values were generated using the inbuilt SPSS multiple imputation procedure, following recommendations of Graham [[5](#_ENREF_5)]. As the cause of data missingness is known and can be accounted for in an imputation procedure, data may be considered missing at random. This situation is similar to that of planned missingness designs [[6](#_ENREF_6), [7](#_ENREF_7)], wherein random sections of a cohort are asked subsets of questions for purposes of maximising the amount of information derived while reducing survey length (by imputing missing values based on the observed relationships between variables). The overlapping questions allow researchers to use imputation to estimate the values of the omitted data as the structure of the underlying correlation matrix can be derived, providing estimates of the associations between all model variables. The mental health domain items were predicted in the equation and predictor variables included: time (baseline vs. follow-up), cohort (ARMHS vs. HCS), age and gender, education (completed 12 years of education vs. not completed 12 years education) and marital status (in a married or defacto relationship vs. not in a married or defacto relationship), as well as all available item responses to the AQoL-6D and Kessler 10 (K10) assessment of psychological distress. Participants included in the imputation procedure had no more than 25% missing data on imputation model variables. Detailed descriptions of the imputation analyses, including AQoL-6D item responses and observed floor and ceiling effects, are presented in Table S2. Items relating to independent living and relationships domains had the poorest response, with the item relating to close and intimate relationships having the poorest response – this is in line with responses from the construction sample [[8](#_ENREF_8)].

## Table S2 Missing value analyses and description of observed item values (N = 8,700)

|  | | | | Item characteristics | | | | |  |  |
| --- | --- | --- | --- | --- | --- | --- | --- | --- | --- | --- |
| Variable | N missing values | % values missing | N observed values | Mean | SD | Median | Min | Max | Floor | Ceiling |
| Time | 0 |  | 8700 | . | . | . | . | . |  |  |
| Cohort | 0 |  | 8700 | . | . | . | . | . |  |  |
| Gender | 0 |  | 8700 | . | . | . | . | . |  |  |
| Age | 18 ^a^ | 0.2% | 8682 | . | . | . | . | . |  |  |
| Education (2 levels) | 346 ^a^ | 4.0% | 8354 | . | . | . | . | . |  |  |
| Marital status (2 levels) | 114 ^a^ | 1.3% | 8586 | . | . | . | . | . |  |  |
| **Kessler 10 (K-10) Items**: |  |  |  |  |  |  |  |  |  |  |
| K10 1 | 44 ^a^ | 0.5% | 8656 | 1.98 | 0.98 | 2 | 1 | 5 | 38.9% | 1.4% |
| K10 2 | 87 ^a^ | 1.0% | 8613 | 1.58 | 0.78 | 1 | 1 | 5 | 57.5% | 0.5% |
| K10 4 | 38 ^a^ | 0.4% | 8657 | 1.12 | 0.42 | 1 | 1 | 5 | 91.4% | 0.2% |
| K10 3 | 43 ^a^ | 0.5% | 8662 | 1.24 | 0.62 | 1 | 1 | 5 | 83.0% | 0.4% |
| K10 5 | 44 ^a^ | 0.5% | 8656 | 1.52 | 0.76 | 1 | 1 | 5 | 61.1% | 0.5% |
| K10 6 | 44 ^a^ | 0.5% | 8656 | 1.23 | 0.58 | 1 | 1 | 5 | 83.5% | 0.3% |
| K10 7 | 46 ^a^ | 0.5% | 8654 | 1.50 | 0.79 | 1 | 1 | 5 | 64.7% | 0.6% |
| K10 8 | 46 ^a^ | 0.5% | 8654 | 1.66 | 0.89 | 1 | 1 | 5 | 55.4% | 1.3% |
| K10 9 | 29 ^a^ | 0.3% | 8671 | 1.23 | 0.58 | 1 | 1 | 5 | 84.1% | 0.3% |
| K10 10 | 37 ^a^ | 0.4% | 8663 | 1.20 | 0.59 | 1 | 1 | 5 | 86.7% | 0.5% |
| **AQoL-6D Items**: |  |  |  |  |  |  |  |  |  |  |
| aq1 (Household tasks) | 101 ^a^ | 1.2% | 8599 | 1.74 | 0.85 | 2 | 1 | 5 | 45.3% | 0.8% |
| aq2 (Getting around) | 87 ^a^ | 1.0% | 8613 | 1.71 | 0.98 | 1 | 1 | 6 | 53.0% | 0.8% |
| aq3 (Walking) | 103 ^a^ | 1.2% | 8597 | 2.29 | 0.97 | 2 | 1 | 6 | 22.1% | 0.1% |
| aq4 (Self-care) | 44 ^a^ | 0.5% | 8656 | 1.34 | 0.62 | 1 | 1 | 5 | 72.7% | 0.1% |
| aq5 (Intimate relationships) | 299 ^a^ | 3.4% | 8401 | 1.80 | 0.76 | 2 | 1 | 5 | 37.0% | 0.7% |
| aq6 (Health & family) | 98 ^a^ | 1.1% | 8602 | 1.20 | 0.47 | 1 | 1 | 4 | 82.6% | 0.2% |
| aq7 (Health & community) | 188 ^a^ | 2.2% | 8512 | 1.28 | 0.64 | 1 | 1 | 4 | 80.1% | 2.3% |
| aq8 (Despair) | 2147 (20) | 24.7% (0.3%) | 6553 | 1.44 | 0.76 | 1 | 1 | 5 | 69.3% | 0.2% |
| aq9 (Worry) | 2146 (19) | 24.7% (0.3%) | 6554 | 1.94 | 0.82 | 2 | 1 | 5 | 30.3% | 0.5% |
| aq10 (Sadness) | 2143 (16) | 24.6% (0.2%) | 6557 | 2.22 | 0.72 | 2 | 1 | 5 | 13.8% | 0.6% |
| aq11 (Agitation) | 2150 (23) | 24.7% (0.3%) | 6550 | 2.29 | 0.59 | 2 | 1 | 5 | 5.6% | 0.1% |
| aq12 (Energy) | 36 ^a^ | 0.4% | 8664 | 2.47 | 0.79 | 2 | 1 | 5 | 5.4% | 1.1% |
| aq13 (Control) | 27 ^a^ | 0.3% | 8673 | 1.83 | 0.70 | 2 | 1 | 5 | 30.8% | 0.4% |
| aq14 (Coping) | 26 ^a^ | 0.3% | 8674 | 1.86 | 0.57 | 2 | 1 | 5 | 22.8% | 0.2% |
| aq15 (Pain frequency) | 66 ^a^ | 0.8% | 8634 | 1.68 | 1.01 | 1 | 1 | 4 | 63.0% | 9.4% |
| aq16 (Pain severity) | 82 ^a^ | 0.9% | 8618 | 1.76 | 0.59 | 2 | 1 | 4 | 32.0% | 0.3% |
| aq17 (Pain impact) | 43 ^a^ | 0.5% | 8657 | 2.15 | 0.97 | 2 | 1 | 5 | 28.4% | 1.7% |
| aq18 (Vision) | 49 ^a^ | 0.6% | 8651 | 2.28 | 0.65 | 2 | 1 | 6 | 9.9% | 0.0% |
| aq19 (Hearing) | 51 ^a^ | 0.6% | 8649 | 2.32 | 0.80 | 2 | 1 | 6 | 16.3% | 0.0% |
| aq20 (Communication) | 78 ^a^ | 0.9% | 8622 | 1.16 | 0.44 | 1 | 1 | 4 | 86.5% | 0.3% |
| **Overall N** | **10900** |  | **302300** |  |  |  |  |  |  |  |
| **% missing data** | **3.48%** |  |  |  |  |  |  |  |  |  |

Note. Bracketed values indicate the number and % of observations missing when excluding baseline ARMHS observations (N = 2127). ^a^ = missing values were imputed for internal purposes only. Cells shaded in grey indicate items with a high proportion of missing values (at or above 1% of participants); Ceiling = % observations with highest possible score on item; Floor = % observations with lowest possible score on item.

Overall, 3.48% of model data were imputed including imputations performed for the SPSS program’s internal calculations. Supplementary Table S3 displays the mean and SD of the point estimate for each AQoL mental health domain item in the observed data, in each of the five datasets, both overall and imputed sets. There was little variation in the value and variability of point estimates for mental health items between observed and imputed data sets (Table S3). Though the item means of imputed values are somewhat lower, this decrease is consistent with the observed ARMHS follow-up values (item 8, mean = 1.29; item 9, mean = 1.89; item 10, mean = 2.10, and; item 11, mean = 2.22) and in part reflects the fact that the overall ARMHS cohort was younger than the HCS cohort.

When restricted to imputed data, imputed values were somewhat lower than the overall data set values, a reduction consistent with all other AQoL-6D item responses at ARMHS baseline, as well as responses to mental health items by ARMHS participants at follow-up.

## Table S3 Estimates for AQoL mental health domain items over 5 imputations

|  |  | Overall (observed and imputed) values by set | | | | | |  | Imputed values by set | | | | | | |
| --- | --- | --- | --- | --- | --- | --- | --- | --- | --- | --- | --- | --- | --- | --- | --- |
|  |  | 1 | 2 | 3 | 4 | 5 | Pooled estimate |  | 1 | 2 | 3 | 4 | 5 | Pooled estimate | max-min |
| Mean | AQoL 8 | 1.37 | 1.38 | 1.38 | 1.38 | 1.38 | 1.38 |  | 1.17 | 1.21 | 1.21 | 1.20 | 1.23 | 1.20 | 0.06 |
|  | AQoL 9 | 1.93 | 1.94 | 1.92 | 1.92 | 1.92 | 1.93 |  | 1.90 | 1.93 | 1.88 | 1.87 | 1.87 | 1.89 | 0.06 |
|  | AQoL 10 | 2.17 | 2.18 | 2.16 | 2.17 | 2.17 | 2.17 |  | 2.02 | 2.04 | 1.99 | 2.01 | 2.00 | 2.01 | 0.05 |
|  | AQoL 11 | 2.24 | 2.25 | 2.24 | 2.25 | 2.25 | 2.25 |  | 2.09 | 2.10 | 2.07 | 2.12 | 2.10 | 2.10 | 0.05 |
| SD | AQoL 8 | 0.77 | 0.76 | 0.76 | 0.77 | 0.76 | 0.76 |  | 0.78 | 0.76 | 0.74 | 0.78 | 0.76 | 0.76 | 0.04 |
|  | AQoL 9 | 0.83 | 0.83 | 0.82 | 0.83 | 0.83 | 0.83 |  | 0.84 | 0.83 | 0.80 | 0.84 | 0.84 | 0.83 | 0.04 |
|  | AQoL 10 | 0.73 | 0.72 | 0.73 | 0.73 | 0.73 | 0.73 |  | 0.75 | 0.71 | 0.72 | 0.73 | 0.73 | 0.73 | 0.03 |
|  | AQoL 11 | 0.61 | 0.61 | 0.60 | 0.60 | 0.60 | 0.60 |  | 0.61 | 0.62 | 0.59 | 0.61 | 0.61 | 0.61 | 0.03 |

In light of the small proportion of data imputed and the small variability between imputed datasets, we examined whether the analytic properties of AQoL items would be perturbed if a single imputed dataset was used for estimation. Internal consistency of the AQoL mental health domain items within single and pooled imputations of baseline ARMHS data, as well as within each of the observed data sets, were examined using Cronbach’s α. Analyses suggest a high level of internal item consistency across baseline ARMHS single (α = .80) and pooled (α = .80) estimates, which was consistent with that observed in the follow-up ARMHS (α = .78), baseline HCS (α = .80) and follow-up HCS (α = .79) sets.

To examine whether associations between variables were influenced by the utilization of single vs. multiple imputation sets, a summed total of the mental health items was regressed onto key predictors within both single and pooled ARMHS baseline imputation sets (Table S4). Observed values at ARMHS follow-up and at HCS baseline and follow-up were examined to determine the expected size and variability of associations. Associations between a summed total of mental health items and selected indicators were highly consistent between single and multiple imputation sets, as well as being similar to follow-up ARMHS associations (Table S4), suggesting that the single imputation set provided adequate representation of the analytic properties of the mental health domain. Thus, a single imputation set generated by a multiple imputation procedure provided satisfactory replacement for AQoL-6D mental health item values and this data was used to replace missing mental health item data for persons with adequate imputation model data at ARMHS baseline.

## Table S4 Comparison of stability of association (β) of a summed total of mental health items regressed onto selected predictors across pooled and single set estimates and with observed associations at HCS baseline and ARMHS and HCS follow-up.

|  | ARMHS | | |  | HCS | |
| --- | --- | --- | --- | --- | --- | --- |
| Predictor | Baseline  single  imputation | Baseline  pooled  imputation | Follow-up observed |  | Baseline observed | Follow-up observed |
| Age | -.16 | -.16 | -.17 |  | -.06 | -.01^ns^ |
|  | (2127) | (2127) | (1227) |  | (3119) | (2151) |
| Female | .06 | .09 | .16 |  | .11 | .12 |
|  | (2127) | (2127) | (1227) |  | (3137) | (2151) |
| Education | .02^ns^ | .02^ns^ | .00^ns^ |  | -.07 | -.06 |
|  | (1998) | (1998) | (1161) |  | (3053) | (2090) |
| Married/defacto | -.15 | -.13 | -.16 |  | -.08 | -.08 |
|  | (2117) | (2117) | (1221) |  | (3062) | (2132) |
| Kessler 10 | .71 | .71 | .72 |  | .72 | .68 |
|  | (2120) | (2120) | (1225) |  | (3125) | (2143) |

All *p* < .01 unless marked as non-significant ^(ns)^; cases in analyses presented as (N).

# Table S5 Raw and standardized weights applied to items to created weighted domain scores

| **Independent living** | **aq1** | **aq2** | **aq3** | **aq4** |
| --- | --- | --- | --- | --- |
| Raw | .156 | .309 | .140 | .202 |
| Standardized | .193 | .383 | .173 | .250 |
| **Relationships** | **aq5** | **aq6** | **aq7** |  |
| Raw | .026 | .300 | .147 |  |
| Standardized | .055 | .634 | .311 |  |
| **Mental health** | **aq8** | **aq9** | **aq10** | **aq11** |
| Raw | .150 | .143 | .145 | .101 |
| Standardized | .278 | .265 | .269 | .187 |
| **Coping** | **aq12** | **aq13** | **aq14** |  |
| Raw | .083 | .278 | .295 |  |
| Standardized | .127 | .424 | .450 |  |
| **Pain** | **aq15** | **aq16** | **aq17** |  |
| Raw | .232 | .380 | .369 |  |
| Standardized | .236 | .387 | .376 |  |
| **Senses** | **aq18** | **aq19** | **aq20** |  |
| Raw | .054 | .176 | .116 |  |
| Standardized | .156 | .509 | .335 |  |

# Table S6 Correlation Matrixes from CFA and SEM analyses

## Subscale CFA

### Independent living

| ROWTYPE_ | VARNAME_ | aq1 | aq2 | aq3 | aq4 |
| --- | --- | --- | --- | --- | --- |
| MEAN |  | 1.716488 | 1.674542 | 2.255338 | 1.319267 |
| STDDEV |  | 0.828464 | 0.945549 | 0.95402 | 0.600334 |
| N |  | 7915 | 7915 | 7915 | 7915 |
| CORR | aq1 | 1 | 0.643551 | 0.568182 | 0.573525 |
| CORR | aq2 | 0.643551 | 1 | 0.672327 | 0.645861 |
| CORR | aq3 | 0.568182 | 0.672327 | 1 | 0.523268 |
| CORR | aq4 | 0.573525 | 0.645861 | 0.523268 | 1 |

### Relationships

| ROWTYPE_ | VARNAME_ | aq5 | aq6 | aq7 |
| --- | --- | --- | --- | --- |
| MEAN |  | 1.794315 | 1.189387 | 1.265319 |
| STDDEV |  | 0.75917 | 0.452593 | 0.617211 |
| N |  | 7915 | 7915 | 7915 |
| CORR | aq5 | 1 | 0.257178 | 0.232978 |
| CORR | aq6 | 0.257178 | 1 | 0.599477 |
| CORR | aq7 | 0.232978 | 0.599477 | 1 |

### Mental health

| ROWTYPE_ | VARNAME_ | aq8 | aq9 | aq10 | aq11 |
| --- | --- | --- | --- | --- | --- |
| MEAN |  | 1.351975 | 1.915712 | 2.160005 | 2.239618 |
| STDDEV |  | 0.751582 | 0.820265 | 0.7211 | 0.601222 |
| N |  | 7915 | 7915 | 7915 | 7915 |
| CORR | aq8 | 1 | 0.572931 | 0.53039 | 0.412159 |
| CORR | aq9 | 0.572931 | 1 | 0.531175 | 0.42846 |
| CORR | aq10 | 0.53039 | 0.531175 | 1 | 0.458308 |
| CORR | aq11 | 0.412159 | 0.42846 | 0.458308 | 1 |

### Coping

| ROWTYPE_ | VARNAME_ | aq12 | aq13 | aq14 |
| --- | --- | --- | --- | --- |
| MEAN |  | 2.447252 | 1.8235 | 1.850284 |
| STDDEV |  | 0.777229 | 0.689136 | 0.563571 |
| N |  | 7915 | 7915 | 7915 |
| CORR | aq12 | 1 | 0.409263 | 0.395207 |
| CORR | aq13 | 0.409263 | 1 | 0.603148 |
| CORR | aq14 | 0.395207 | 0.603148 | 1 |

### Pain

| ROWTYPE_ | VARNAME_ | aq15 | aq16 | aq17 |
| --- | --- | --- | --- | --- |
| MEAN |  | 1.64144 | 1.743651 | 2.121036 |
| STDDEV |  | 0.983334 | 0.582673 | 0.953827 |
| N |  | 7915 | 7915 | 7915 |
| CORR | aq15 | 1 | 0.597757 | 0.657907 |
| CORR | aq16 | 0.597757 | 1 | 0.653103 |
| CORR | aq17 | 0.657907 | 0.653103 | 1 |

### Senses

| ROWTYPE_ | VARNAME_ | aq18 | aq19 | aq20 |
| --- | --- | --- | --- | --- |
| MEAN |  | 2.268478 | 2.306507 | 1.154138 |
| STDDEV |  | 0.647938 | 0.795998 | 0.427739 |
| N |  | 7915 | 7915 | 7915 |
| CORR | aq18 | 1 | 0.255695 | 0.155678 |
| CORR | aq19 | 0.255695 | 1 | 0.326979 |
| CORR | aq20 | 0.155678 | 0.326979 | 1 |

## Overall CFA

| ROWTYPE_ | VARNAME_ | AQoL_IL_ML_FS | AQoL_REL_ML_FS | AQoL_MH_ML_FS | AQoL_COP_ML_FS | AQoL_PAI_ML_FS | AQoL_SEN_ML_FS |
| --- | --- | --- | --- | --- | --- | --- | --- |
| MEAN |  | 1.692621984 | 1.246273026 | 1.883362878 | 1.916592798 | 1.859682502 | 1.914530512 |
| STDDEV |  | 0.717653108 | 0.44369042 | 0.584205785 | 0.540574531 | 0.714865657 | 0.508257986 |
| N |  | 7915 | 7915 | 7915 | 7915 | 7915 | 7915 |
| CORR | AQoL_IL_ML_FS | 1 | 0.670408907 | 0.326994839 | 0.395179134 | 0.581681175 | 0.274618887 |
| CORR | AQoL_REL_ML_FS | 0.670408907 | 1 | 0.389997124 | 0.439783394 | 0.476524484 | 0.241296785 |
| CORR | AQoL_MH_ML_FS | 0.326994839 | 0.389997124 | 1 | 0.639818172 | 0.338419376 | 0.188877746 |
| CORR | AQoL_COP_ML_FS | 0.395179134 | 0.439783394 | 0.639818172 | 1 | 0.326382479 | 0.220338368 |
| CORR | AQoL_PAI_ML_FS | 0.581681175 | 0.476524484 | 0.338419376 | 0.326382479 | 1 | 0.220819915 |
| CORR | AQoL_SEN_ML_FS | 0.274618887 | 0.241296785 | 0.188877746 | 0.220338368 | 0.220819915 | 1 |

Note: The last 6 columns refer to the AQoL-6D domains: independent living (IL), relationships (REL), mental health (MH), coping (COP), pain (PAI), senses (SEN).

## MGCFA: Cohort invariance

### ARMHS

| ROWTYPE_ | VARNAME_ | AQoL_IL_ML_FS | AQoL_REL_ML_FS | AQoL_MH_ML_FS | AQoL_COP_ML_FS | AQoL_PAI_ML_FS | AQoL_SEN_ML_FS |
| --- | --- | --- | --- | --- | --- | --- | --- |
| MEAN |  | 1.577748547 | 1.197472886 | 1.781171781 | 1.87458328 | 1.772601033 | 1.789696578 |
| STDDEV |  | 0.67371284 | 0.394625776 | 0.58144364 | 0.548374425 | 0.690587831 | 0.492758997 |
| N |  | 3098 | 3098 | 3098 | 3098 | 3098 | 3098 |
| CORR | AQoL_IL_ML_FS | 1 | 0.634983426 | 0.291675014 | 0.362883059 | 0.570580789 | 0.250496032 |
| CORR | AQoL_REL_ML_FS | 0.634983426 | 1 | 0.409331635 | 0.450503393 | 0.450836921 | 0.189869662 |
| CORR | AQoL_MH_ML_FS | 0.291675014 | 0.409331635 | 1 | 0.665333618 | 0.303196012 | 0.123523327 |
| CORR | AQoL_COP_ML_FS | 0.362883059 | 0.450503393 | 0.665333618 | 1 | 0.313367859 | 0.174764779 |
| CORR | AQoL_PAI_ML_FS | 0.570580789 | 0.450836921 | 0.303196012 | 0.313367859 | 1 | 0.235763075 |
| CORR | AQoL_SEN_ML_FS | 0.250496032 | 0.189869662 | 0.123523327 | 0.174764779 | 0.235763075 | 1 |

Note: The last 6 columns refer to the AQoL-6D domains: independent living (IL), relationships (REL), mental health (MH), coping (COP), pain (PAI), senses (SEN).

### HCS

| ROWTYPE_ | VARNAME_ | AQoL_IL_ML_FS | AQoL_REL_ML_FS | AQoL_MH_ML_FS | AQoL_COP_ML_FS | AQoL_PAI_ML_FS | AQoL_SEN_ML_FS |
| --- | --- | --- | --- | --- | --- | --- | --- |
| MEAN |  | 1.766501557 | 1.277658294 | 1.949085946 | 1.943610754 | 1.91568798 | 1.994816068 |
| STDDEV |  | 0.735187108 | 0.469931103 | 0.576541076 | 0.53381096 | 0.724614342 | 0.501881717 |
| N |  | 4817 | 4817 | 4817 | 4817 | 4817 | 4817 |
| CORR | AQoL_IL_ML_FS | 1 | 0.683962072 | 0.328882148 | 0.409255006 | 0.580212479 | 0.260095771 |
| CORR | AQoL_REL_ML_FS | 0.683962072 | 1 | 0.370749524 | 0.432421138 | 0.48416989 | 0.251161119 |
| CORR | AQoL_MH_ML_FS | 0.328882148 | 0.370749524 | 1 | 0.620865093 | 0.346013088 | 0.193246996 |
| CORR | AQoL_COP_ML_FS | 0.409255006 | 0.432421138 | 0.620865093 | 1 | 0.328534663 | 0.237279331 |
| CORR | AQoL_PAI_ML_FS | 0.580212479 | 0.48416989 | 0.346013088 | 0.328534663 | 1 | 0.189073294 |
| CORR | AQoL_SEN_ML_FS | 0.260095771 | 0.251161119 | 0.193246996 | 0.237279331 | 0.189073294 | 1 |

Note: The last 6 columns refer to the AQoL-6D domains: independent living (IL), relationships (REL), mental health (MH), coping (COP), pain (PAI), senses (SEN).

## MGCFA: Temporal invariance

### Baseline

| ROWTYPE_ | VARNAME_ | AQoL_IL_ML_FS | AQoL_REL_ML_FS | AQoL_MH_ML_FS | AQoL_COP_ML_FS | AQoL_PAI_ML_FS | AQoL_SEN_ML_FS |
| --- | --- | --- | --- | --- | --- | --- | --- |
| MEAN |  | 1.690323753 | 1.262719975 | 1.862599092 | 1.921177992 | 1.891414289 | 1.905920345 |
| STDDEV |  | 0.728453525 | 0.457905283 | 0.596877479 | 0.54556576 | 0.731353045 | 0.510281941 |
| N |  | 4871 | 4871 | 4871 | 4871 | 4871 | 4871 |
| CORR | AQoL_IL_ML_FS | 1 | 0.679145612 | 0.320664373 | 0.395820493 | 0.605886867 | 0.266053829 |
| CORR | AQoL_REL_ML_FS | 0.679145612 | 1 | 0.405303773 | 0.456557684 | 0.496822021 | 0.231792255 |
| CORR | AQoL_MH_ML_FS | 0.320664373 | 0.405303773 | 1 | 0.644331109 | 0.342024606 | 0.195640378 |
| CORR | AQoL_COP_ML_FS | 0.395820493 | 0.456557684 | 0.644331109 | 1 | 0.334798095 | 0.235466708 |
| CORR | AQoL_PAI_ML_FS | 0.605886867 | 0.496822021 | 0.342024606 | 0.334798095 | 1 | 0.240294273 |
| CORR | AQoL_SEN_ML_FS | 0.266053829 | 0.231792255 | 0.195640378 | 0.235466708 | 0.240294273 | 1 |

Note: The last 6 columns refer to the AQoL-6D domains: independent living (IL), relationships (REL), mental health (MH), coping (COP), pain (PAI), senses (SEN).

### Follow-up

| ROWTYPE_ | VARNAME_ | AQoL_IL_ML_FS | AQoL_REL_ML_FS | AQoL_MH_ML_FS | AQoL_COP_ML_FS | AQoL_PAI_ML_FS | AQoL_SEN_ML_FS |
| --- | --- | --- | --- | --- | --- | --- | --- |
| MEAN |  | 1.696299606 | 1.219954665 | 1.916589028 | 1.909255585 | 1.808905388 | 1.928308476 |
| STDDEV |  | 0.700126706 | 0.41867559 | 0.561838115 | 0.532497258 | 0.684725866 | 0.504780476 |
| N |  | 3044 | 3044 | 3044 | 3044 | 3044 | 3044 |
| CORR | AQoL_IL_ML_FS | 1 | 0.65767269 | 0.338657934 | 0.39430257 | 0.541959365 | 0.289050248 |
| CORR | AQoL_REL_ML_FS | 0.65767269 | 1 | 0.370042686 | 0.410320908 | 0.434756956 | 0.262271427 |
| CORR | AQoL_MH_ML_FS | 0.338657934 | 0.370042686 | 1 | 0.635426012 | 0.341476358 | 0.175416163 |
| CORR | AQoL_COP_ML_FS | 0.39430257 | 0.410320908 | 0.635426012 | 1 | 0.311634405 | 0.196054433 |
| CORR | AQoL_PAI_ML_FS | 0.541959365 | 0.434756956 | 0.341476358 | 0.311634405 | 1 | 0.191761111 |
| CORR | AQoL_SEN_ML_FS | 0.289050248 | 0.262271427 | 0.175416163 | 0.196054433 | 0.191761111 | 1 |

Note: The last 6 columns refer to the AQoL-6D domains: independent living (IL), relationships (REL), mental health (MH), coping (COP), pain (PAI), senses (SEN).

# Glossary

- *r*_icc_: Intra-class correlation coefficient (absolute).
- α: Cronbach’s alpha is an index of internal consistency (or item reliability) that uses item correlations to assess a common construct.
- H: coefficient H is a reliability index that uses model parameters to determine the reliability with which items assess a latent construct.
- CFA: confirmatory factor analyses.
- SMC: Squared multiple correlation (interpretation similar to R^2^ in multiple regression analyses).
- MGCFA: multi group confirmatory factor analyses.
- Measurement invariance: indicators display consistent associations with latent factors across groups.
- Structural invariance: latent variables display consistent measurement properties and associations among themselves across groups.
- Configural invariance: an assessment of whether the indicators are associated with the same latent factors across groups, phases or other data subsets. Models assessing configural invariance constrain the model so that indicators load on the same latent factor across groups. A form of measurement invariance.
- Metric invariance: an assessment of whether the indicators associated with latent factors have the same strength and direction across groups, phases or other data subsets. Models assessing metric invariance constrain model so that the factor loadings of a latent variable with its indicators are equal across groups. A form of measurement invariance.
- Variance/co-variance invariance: an assessment of whether the latent factors represent the same range of values and are related to one another with the same strength across groups, phases or other data subsets. Models assessing variance/co-variance invariance constrain the model so that the variance of latent variables is equal across groups and the correlations between latent variables are equal across groups. A form of structural invariance.
- AQoL-6D Item: single question for which the AQoL-6D has 4-6 response options.
- AQoL-6D Domain: group of questions targeting the same area of quality of life.
- AQoL-6D Factor(s): higher-order factor(s) tapping common constructs across one or more domains (also labelled as Summary Scores).
- AQoL-6D total score: a single value representing respondent quality of life.

# Additional References

1. Hayes A, Clarke P, Lung T: **Change in bias in self-reported body mass index in Australia between 1995 and 2008 and the evaluation of correction equations.** *Population Health Metrics* 2011, **9:**53.

2. Stommel M, Osier N: **Temporal changes in bias of body mass index scores based on self-reported height and weight.** *Int J Obes* 2012.

3. Australian Bureau of Statistics: *Body Mass Index by age and sex – Australia (4364.0.55.001). Australian Health Survey: First Results, 2011-12.*  Australian Government. Canberra; 2012.

4. Little RJA, Rubin DB: *Statistical Analysis with Missing Data.* 2nd edn. New York: John Wiley; 2002.

5. Graham JW: **Missing Data Analysis: Making It Work in the Real World.** *Annu Rev Psychol* 2009, **60:**549-576.

6. Graham J, Taylor B, Olchowski A, Cumsille P: **Planned missing data designs in psychological research.** *Psychol Methods* 2006, **11:**323-343.

7. Raghunathan TE, Grizzle JE: **A Split Questionnaire Survey Design.** *Journal of the American Statistical Association* 1995, **90:**54-63.

8. Day N, Richardson J, Hawthorne G: *Modelling health related quality of life for the AQoL-6D Research paper 51.*  Melbourne: Centre for Health Economics, Monash University. 2010.
