# Supplementary material for: Construct validity of the Assessment of Quality of Life - 6D (AQoL-6D) in community samples
Source: Health Qual Life Outcomes. 2013 Apr 17;11:61. doi: 10.1186/1477-7525-11-61 (PMC3639231; doi:10.1186/1477-7525-11-61)
Supplement: Additional file 2 — Appendix 1 AQoL-6D items and scoring. [file 1477-7525-11-61-S2.docx]

**Assessment of Quality of Life (AQoL-6D)^#^**

**Please read each question or statement and tick the box next to the response that best fits your current situation.**

| **Q1** | **How much help do you need with household tasks (e.g. preparing food, cleaning the house or gardening):** |
| --- | --- |
| □ 1 | I can do all these tasks very quickly and efficiently without any help |
| □ 2 | I can do these tasks relatively easily without help |
| □ 3 | I can do these tasks only very slowly without help |
| □ 4 | I cannot do most of these tasks unless I have help |
| □ 5 | I can do none of these tasks by myself |
| **Q2** | **Thinking about how easy or difficult it is for you to get around by yourself outside your house (e.g. shopping, visiting):** |
| □ 1 | Getting around is enjoyable and easy |
| □ 2 | I have no difficulty getting around outside my house |
| □ 3 | A little difficulty |
| □ 4 | Moderate difficulty |
| □ 5 | A lot of difficulty |
| □ 6 | I cannot get around unless somebody is there to help me |
| **Q3** | **Thinking about how well you can walk:** |
| □ 1 | I find walking or running very easy |
| □ 2 | I have no real difficulty with walking or running |
| □ 3 | I find walking or running slightly difficult. I cannot run to catch a tram or train, I find walking uphill difficult |
| □ 4 | Walking is difficult for me. I walk short distances only, I have difficulty walking up stairs |
| □ 5 | I have great difficulty walking. I cannot walk without a walking stick or frame, or someone to help me |
| □ 6 | I am bedridden |
| **Q4** | **Thinking about washing yourself, toileting, dressing, eating or looking after your appearance:** |
| □ 1 | These tasks are very easy for me |
| □ 2 | I have no real difficulty in carrying out these tasks |
| □ 3 | I find some of these tasks difficult, but I manage to do them on my own |
| □ 4 | Many of these tasks are difficult, and I need help to do them |
| □ 5 | I cannot do these tasks by myself at all |
| **Q5** | **My close and intimate relationships (including any sexual relationships) make me:** |
| □ 1 | Very happy |
| □ 2 | Generally happy |
| □ 3 | Neither happy nor unhappy |
| □ 4 | Generally unhappy |
| □ 5 | Very unhappy |
| **Q6** | **Thinking about your health and your relationships with your family:** |
| □ 1 | My role in the family is unaffected by my health |
| □ 2 | There are some parts of my family role I cannot carry out |
| □ 3 | There are many parts of my family role I cannot carry out |
| □ 4 | I cannot carry out any part of my family role |
| **Q7** | **Thinking about your health and your role in your community (that is to say, neighbourhood, sporting, work, church or cultural groups):** |
| □ 1 | My role in the community is unaffected by my health |
| □ 2 | There are some parts of my community role I cannot carry out |
| □ 3 | There are many parts of my community role I cannot carry out |
| □ 4 | I cannot carry out any part of my community role |

Office Use Only

| **Independent Living (Q1+Q2+Q3+Q4)/4 =** |  |
| --- | --- |
| **Relationships (Q5+Q6+Q7)/3 =** |  |

| **Q8** | **How often did you feel in despair over the last seven days?** |
| --- | --- |
| □ 1 | Never |
| □ 2 | Occasionally |
| □ 3 | Sometimes |
| □ 4 | Often |
| □ 5 | All the time |
| **Q9** | **And still thinking about the last seven days, how often did you feel worried?** |
| □ 1 | Never |
| □ 2 | Occasionally |
| □ 3 | Sometimes |
| □ 4 | Often |
| □ 5 | All the time |
| **Q10** | **How often do you feel sad?** |
| □ 1 | Never |
| □ 2 | Rarely |
| □ 3 | Some of the time |
| □ 4 | Usually |
| □ 5 | Nearly all the time |
| **Q11** | **When you think about whether you are calm and tranquil or agitated: I am** |
| □ 1 | Always calm and tranquil |
| □ 2 | Usually calm and tranquil |
| □ 3 | Sometimes calm and tranquil, sometimes agitated |
| □ 4 | Usually agitated |
| □ 5 | Always agitated |
| **Q12** | **Thinking about how much energy you have to do the things you want to do: I am** |
| □ 1 | Always full of energy |
| □ 2 | Usually full of energy |
| □ 3 | Occasionally energetic |
| □ 4 | Usually tired and lacking energy |
| □ 5 | Always tired and lacking energy |
| **Q13** | **How often do you feel in control of your life?** |
| □ 1 | Always |
| □ 2 | Mostly |
| □ 3 | Sometimes |
| □ 4 | Only occasionally |
| □ 5 | Never |
| **Q14** | **How much do you feel you can cope with life’s problems?** |
| □ 1 | Completely |
| □ 2 | Mostly |
| □ 3 | Partly |
| □ 4 | Very little |
| □ 5 | Not at all |

Office Use Only

| **Mental Health (Q8+Q9+Q10+Q11)/4 =** |  |
| --- | --- |
| **Coping (Q12+Q13+Q14)/3 =** |  |

| **Q15** | **Thinking about how often you experience serious pain: I experience it** |
| --- | --- |
| □ 1 | Very rarely |
| □ 2 | Less than once a week |
| □ 3 | Three to four times a week |
| □ 4 | Most of the time |
| **Q16** | **How much pain or discomfort do you experience:** |
| □ 1 | None at all |
| □ 2 | I have moderate pain |
| □ 3 | I suffer from severe pain |
| □ 4 | I suffer unbearable pain |
| **Q17** | **How often does pain interfere with your usual activities?** |
| □ 1 | Never |
| □ 2 | Rarely |
| □ 3 | Sometimes |
| □ 4 | Often |
| □ 5 | Always |
| **Q18** | **Thinking about your vision (using your glasses or contact lenses if needed):** |
| □ 1 | I have excellent sight |
| □ 2 | I see normally |
| □ 3 | I have some difficulty focusing on things, or I do not see them sharply. For example: small print, a newspaper or seeing objects in the distance. |
| □ 4 | I have a lot of difficulty seeing things. My vision is blurred. I can see just enough to get by with. |
| □ 5 | I only see general shapes. I need a guide to move around |
| □ 6 | I am completely blind |
| **Q19** | **Thinking about your hearing (using your hearing aid if needed):** |
| □ 1 | I have excellent hearing |
| □ 2 | I hear normally |
| □ 3 | I have some difficulty hearing or I do not hear clearly. I have trouble hearing softly-spoken people or when there is background noise. |
| □ 4 | I have difficulty hearing things clearly. For example: Often I do not understand what is said. I usually do not take part in conversations because I cannot hear what is said. |
| □ 5 | I hear very little indeed. I cannot fully understand loud voices speaking directly to me. |
| □ 6 | I am completely deaf |
| **Q20** | **When you communicate with others (e.g. by talking, listening, writing or signing):** |
| □ 1 | I have no trouble speaking to them or understanding what they are saying |
| □ 2 | I have some difficulty being understood by people who do not know me. I have no trouble understanding what others are saying to me. |
| □ 3 | I am understood only by people who know me well. I have great trouble understanding what others are saying to me. |
| □ 4 | I cannot adequately communicate with others |

Office Use Only

| **Pain (Q15+Q16+Q17)/3 =** |  |
| --- | --- |
| **Senses (Q18+Q19+Q20)/3 =** |  |

| **Psychological (Mental Health + Coping)/2 =** |  |
| --- | --- |
| **Physical (Independent Living + Relationships + Pain + Senses)/4 =** |  |

| **Total score (Psychological + Physical)/2 =** |  |
| --- | --- |
